# Direct imaging of glycans in Arabidopsis roots via click labeling of metabolically incorporated azido-monosaccharides

**DOI:** 10.1186/s12870-016-0907-0

**Published:** 2016-10-10

**Authors:** Jorin Hoogenboom, Nathalja Berghuis, Dario Cramer, Rene Geurts, Han Zuilhof, Tom Wennekes

**Affiliations:** 1 Laboratory of Organic Chemistry, Wageningen University, Stippeneng 4, 6708 WE Wageningen, The Netherlands; 2 Department of Chemical Biology and Drug Discovery, Utrecht Institute for Pharmaceutical Sciences and Bijvoet Center for Biomolecular Research, Utrecht University, Utrecht, The Netherlands; 3 Department of Plant Science, Laboratory of Molecular Biology, Wageningen University, Droevendaalsesteeg 1, 6708 PB Wageningen, The Netherlands

**Keywords:** Click chemistry, *Arabidopsis thaliana*, Cell wall, Glycans, l-Arabinofuranose, d-Glucosamine, d-Galactosamine, l-Fucose, Metabolic oligosaccharide engineering

## Abstract

**Background:**

Carbohydrates, also called glycans, play a crucial but not fully understood role in plant health and development. The non-template driven formation of glycans makes it impossible to image them in vivo with genetically encoded fluorescent tags and related molecular biology approaches. A solution to this problem is the use of tailor-made glycan analogs that are metabolically incorporated by the plant into its glycans. These metabolically incorporated probes can be visualized, but techniques documented so far use toxic copper-catalyzed labeling. To further expand our knowledge of plant glycobiology by direct imaging of its glycans via this method, there is need for novel click-compatible glycan analogs for plants that can be bioorthogonally labelled via copper-free techniques.

**Results:**

Arabidopsis seedlings were incubated with azido-containing monosaccharide analogs of *N*-acetylglucosamine, *N*-acetylgalactosamine, l-fucose, and l-arabinofuranose. These azido-monosaccharides were metabolically incorporated in plant cell wall glycans of Arabidopsis seedlings. Control experiments indicated active metabolic incorporation of the azido-monosaccharide analogs into glycans rather than through non-specific absorption of the glycan analogs onto the plant cell wall. Successful copper-free labeling reactions were performed, namely an inverse-electron demand Diels-Alder cycloaddition reaction using an incorporated *N*-acetylglucosamine analog, and a strain-promoted azide-alkyne click reaction. All evaluated azido-monosaccharide analogs were observed to be non-toxic at the used concentrations under normal growth conditions.

**Conclusions:**

Our results for the metabolic incorporation and fluorescent labeling of these azido-monosaccharide analogs expand the possibilities for studying plant glycans by direct imaging. Overall we successfully evaluated five azido-monosaccharide analogs for their ability to be metabolically incorporated in Arabidopsis roots and their imaging after fluorescent labeling. This expands the molecular toolbox for direct glycan imaging in plants, from three to eight glycan analogs, which enables more extensive future studies of spatiotemporal glycan dynamics in a wide variety of plant tissues and species. We also show, for the first time in metabolic labeling and imaging of plant glycans, the potential of two copper-free click chemistry methods that are bio-orthogonal and lead to more uniform labeling. These improved labeling methods can be generalized and extended to already existing and future click chemistry-enabled monosaccharide analogs in Arabidopsis.

**Electronic supplementary material:**

The online version of this article (doi:10.1186/s12870-016-0907-0) contains supplementary material, which is available to authorized users.

## Background

All plant cells are covered by a dense layer of carbohydrates (glycans), called the glycocalyx. It is the glycocalyx that is first encountered by other cells, including microbes. Glycans are also found on more than 50 % of plant proteins as an important post-translational modification that directly influences protein functioning [1]. Hence it is not surprising that glycans play essential roles in a myriad of biological processes in all stages of plant development, such as cell-cell communication [2], control of metabolism, growth, stress response [3] and external signalling, thereby also tied to the rhizosphere [4–6]. Glycans thus play a crucial but not well understood role in plant health and disease. Developing techniques to better study plant glycans and increase our understanding of and control over their role is an essential next step in plant sciences.

Due to the non-template driven formation of glycans it is not possible to use genetically encoded protein-based fluorescent tags to image and study glycans directly. Externally added protein-based probes, usually fluorescently-labeled lectins, image the glycans indirectly and are only able to image glycans exposed on the most outer layer of the cell surface glycocalyx [7, 8].

Another approach, however, exists that allows the direct imaging of plant glycans. Glycans, especially in plants, usually have highly complex and diverse structures containing monosaccharides such as glucose, *N*-acetylglucosamine, galactose, l-arabinose, xylose, l-fucose and 3-deoxy-d-manno-oct-2-ulosonic acid (KDO) [9–11]. Besides their de novo biosynthesis, these monosaccharides and their derivatives can also be recycled by plant cells [12]. Through uptake of extracellular monosaccharides and by intracellular catabolism of complex plant glycans, these monosaccharides can be recycled through the glycan salvage pathways. Using this recycling pathway the monosaccharides again end up in plant cell-surface glycans and its glycoproteins [13]. Glycans and their conjugates are biosynthesized by glycosyltransferases present in the Golgi apparatus and endoplasmic reticulum (ER). The composition and levels of glycans in the glycocalyx and in proteins depends on the presence and levels of these enzymes and their activated monosaccharide donor substrates [12].

**Fig. 1:**
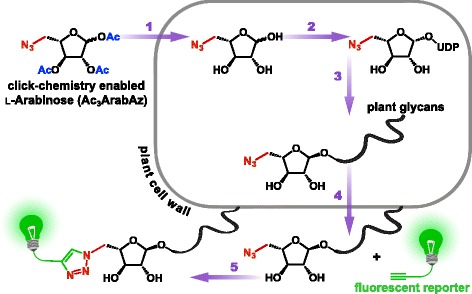
Metabolic labeling of Arabidopsis cell wall-glycans with azido-monosaccharides. Arabidopsis is grown on MS containing an azido-monosaccharide such as Ac_3_ArabAz, which is taken up through the cell wall followed by hydrolysis of the acetyl (Ac) groups by intracellular esterases (1). The resulting ArabAz enters the glycan salvage pathway and is converted to an azido-nucleotide sugar donor (2) that allows its incorporation by glycosyltransferases into plant glycans (3) that end up in plant cell-surface glycans and its glycoproteins (4). Finally, the incorporated glycan can be imaged after a click-reaction with a fluorescent reporter group (5) (see Additional file 14 for high resolution)

The metabolic incorporation of monosaccharide analogs with a latent imaging tag via these pathways would allow for the direct imaging of plant glycans (Fig. 1) [12]. These incorporated monosaccharide analogs can be visualized and studied through a tag that enables click chemistry, which allows for rapid, specific and versatile covalent labeling of plant glycans with a fluorescent reporter molecule [14, 15]. This technique is called Metabolic Oligosaccharide Engineering (MOE) and it has already been widely applied for studying glycobiology in various organisms, with the notable exception of plants [16]. Indeed, only in 2012, the first application of MOE with click-compatible monosaccharide analogs in plants was reported by Anderson et al. in which fucosylated plant glycans were fluorescently imaged in *Arabidopsis thaliana* (Col-0) seedlings [17]. Two other click-compatible monosaccharide analogs were reported recently, namely, 6-deoxy-alkynyl-glucose that incorporates in Arabidopsis root hair tips [18], and 8-azido-8-deoxy-KDO, a probe analogous to KDO that is present in the cell wall pectic polysaccharide, rhamnogalacturonan II [19]. To further expand our knowledge of plant glycobiology by direct imaging of glycans, there is need for click chemistry-compatible glycan analogs for other plant monosaccharides. In addition, the click chemistry compatible glycan analogs in plants documented so far were labeled using toxic copper-labeling, and future applications would benefit from bio-orthogonal copper-free labeling techniques.

**Fig. 2:**
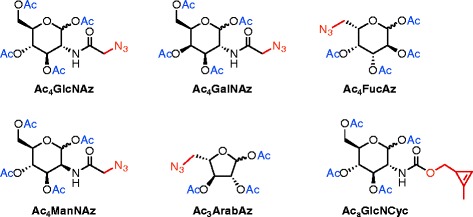
Chemical structure of click chemistry-enabled monosaccharide analogs that were used in this study (see Additional file 14 for high resolution)

We investigated five glycans: *N*-acetyl-d-glucosamine, l-fucose and l-arabinose - which are all known to be present in the glycocalyx of Arabidopsis [11, 20] - and *N*-acetyl-d-galactosamine (GalNAc) and *N*-acetyl-d-mannosamine. While the latter two glycans are not known to be present in plant glycans, it was recently discovered that UDP-GalNAc is present and transported in the ER of Arabidopsis [21], indicating that GalNAc is metabolized by plants. In this technical advance paper we expand the monosaccharide analog toolbox (Fig. 2) for metabolic labeling of glycans in Arabidopsis seedlings. Furthermore, the glycan analogs reported so far in plants use a Cu(I)-catalyzed cycloaddition, however, this is cytotoxic for Arabidopsis [22] and microbes in soils [23] making this method less suitable for long-term and more complex experiments with living plants. Therefore we investigated the possibilities of bio-orthogonal copper-free click reactions in Arabidopsis roots.

## Results and discussion

### Azido-monosaccharides are not toxic at experimental concentrations

To determine if Arabidopsis seedlings behave differently under normal growth conditions when incubated with our non-natural azido-containing monosaccharide analogs (Fig. 2), their toxicity was evaluated. Earlier reports of metabolic labeling of plant seedlings with monosaccharide analogs have evaluated toxicity by measuring the root length of 8-day old seedlings on MS plates [22, 24]. This toxicity evaluation exposes plant seedlings for several days to high levels of azido-monosaccharides, while the metabolic incorporation experiments are carried out at similar or lower concentrations in a fraction of that time (typically 4-24 h). Accordingly, seedlings were exposed for 8 days to the azido-monosaccharides at concentrations that were used in the different metabolic incorporation experiments (10, 25 and 100 μM). When compared to seedlings grown on agarose plates with only MS medium, no significant difference was observed (Additional file 1). This shows that azido-monosaccharides do not significantly influence the growth and metabolic processes in Arabidopsis.

### Ac_4_GlcNAz, Ac_3_ArabAz and Ac_4_FucAz are incorporated in root cell walls of differentiating *Arabidopsis*

**Fig. 3:**
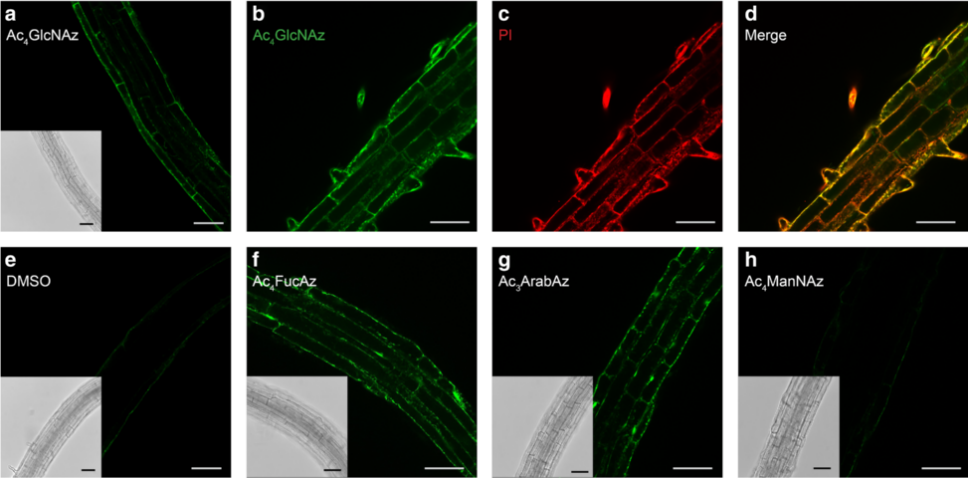
Optical sections of 4 day old Arabidopsis seedling roots incubated for 24 h with azido-monosaccharides. Seedlings were incubated with 25 μM Ac_4_GlcNAz (**a**), followed by labeling through a copper-catalyzed click reaction with Alexa Fluor® 488 alkyne. Seedling roots treated with Alexa Fluor® 488 alkyne-labeled Ac_4_GlcNAz (25 μM, 24 h) (**b**, **d**) were counterstained; Propidium Iodide (PI, 0.05 %) to visualize cell walls (**c**, **d**). Yellow color indicates overlap of the two dyes (**d**). Scale bars = 50 μm. As a control, seedlings were treated with 0.01 % DMSO (**e**). Alternatively, seedlings were incubated with 25 μM Ac_4_FucAz (**f**), 100 μM Ac_3_ArabAz (**g**), or 25 μM Ac_4_ManNAz (**h**) (see Additional file 14 for high resolution)

*N*-acetylglucosamine is commonly present in *N*-glycans of plant cell walls [11, 20] and is important for *N-*glycan formation, since it is the first monosaccharide attached to glycoproteins [25]. Therefore metabolic click-mediated labeling of Arabidopsis cell walls was investigated with a *N*-acetyl-glucosamine analog containing a clickable azide (Ac_4_GlcNAz; Fig. 2). Ac_4_GlcNAz was synthesized according to a procedure of Bertozzi and co-workers [26]. Ac_4_GlcNAz was dissolved in a ½ MS medium and used to incubate four day-old Arabidopsis (Col-0) seedlings. The seedlings were incubated with 10, 25, 50 or 100 μM Ac_4_GlcNAz and control seedlings were incubated with 0.01 % DMSO. After 24 h, seedlings were washed and then transferred for 45 min to a solution containing Alexa Fluor® 488-alkyne and a Click-iT kit solution for the copper-catalyzed labelling. After the labeling and the subsequent washing steps, fluorescence intensity of cell walls was monitored by confocal microscopy. Seedlings incubated with 25 μM Ac_4_GlcNAz showed optimal labeling (Fig. 3a & Additional file 2). Increased labeling could be reached at higher concentrations, but was not required (Additional file 2B). Control seedlings (Fig. 3e) treated with 0.01 % DMSO did not show auto-fluorescence background signals under these conditions and therefore 25 μM was used for further labeling experiments with Ac_4_GlcNAz.

To determine the subcellular localization of Ac_4_GlcNAz, Ac_4_GlcNAz-labeled roots (Fig. 3b) were counterstained with propidium iodide (Fig. 3c) (PI). This revealed that both signals showed overlap (Fig. 3d), indicating a location of Ac_4_GlcNAz in or at the cell walls. During these labeling experiments we focused on studying the transition zone because strong labeling was observed in this region. Directly above this region a decline in labeling was observed, while the meristem zone showed only a slight decrease in labeling.

Encouraged by these results we decided to investigate incorporation and visualization of sugar analogues of l-arabinose and l-fucose. Both of these monosaccharides are commonly found in oligosaccharides of plants. More specifically, l-arabinose - mainly present in Arabidopsis as l-arabinofuranose - is one of the most common *O-*glycan sugars and an important constituent of plant cell wall polysaccharides [27–30]. Furthermore, an alkynylated fucose analog was the first successful metabolically incorporated sugar in Arabidopsis cell walls [22]. Hence, we investigated whether the azido-analogues of l-fucose and l-arabinose may be metabolically incorporated into glycans by Arabidopsis seedling roots. To that end, the two corresponding azido analogues of these sugars were synthesized; Ac_3_ArabAz and Ac_4_FucAz (Fig. 2). Ac_4_FucAz was prepared by acetylating commercially available 6-azido-l-fucose, while the novel Ac_3_ArabAz was prepared according to an adapted procedure of 5-azido-d-arabinose by Smellie and co-workers [31]. With both azido-monosaccharides in hand, the feasibility of the incorporations of these compounds was investigated. Similar to the investigation of Ac_4_GlcNAz the optimal incorporation was determined by using different concentrations of Ac_4_FucAz (Additional file 3) and Ac_3_ArabAz (Additional file 4). Clear incorporation of Ac_4_FucAz was observed at the 25 μM range (Fig. 3f), whereas reliable incorporation of Ac_3_ArabAz was only observed after incubation at a concentration of 100 μM (Fig. 3g). This is most likely due to the relatively high abundance of naturally occurring l-arabinose compared to *N*-acetylglucosamine and l-fucose. As such, the relatively high concentration of l-arabinose would compete during incorporation of Ac_3_ArabAz at low concentrations of this probe.

l-Arabinofuranosyl residues are incorporated into plant arabinogalactan from UDP-Ara*f* by glycosyltransferases. This nucleotide sugar donor is believed to be biosynthesized exclusively from the thermodynamically more stable pyranosyl form of the same donor; UDP-Ara*p*. However, ArabAz is not able to convert to its pyranose configuration meaning that the corresponding pyranosyl UDP-ArabAz cannot exist. This raises the question how ArabAz is incorporated. Fincher and coworkers recently reported on the catalytic properties of an UDP-arabinose mutase (UAM) enzyme in Barley that catalyzes the multistep reversible UDP-Ara*p* → UDP-Ara*f* reaction [32]. They state that a key step in this reaction presumably includes cleavage of the arabinosyl residue from UDP-Ara*p*, which allows opening of the pyranosyl-ring, formation of the furanose ring, and reconnection of the arabinofuranosyl residue to the UDP molecule. Similar UDP-mutase enzymes (RGP) have been reported in other plant species, including Arabidopsis [33]. Consequently, ArabAz may be recognized by these enzymes and converted to UDP-ArabAz and thus enable metabolic incorporation.

Next, the results obtained with the three azido-monosaccharides were compared with an azido analog of *N*-acetyl-d-mannosamine; Ac_4_ManNAz (Fig. 3h and Additional file 5). Ac_4_ManNAz differs in only one chiral centre compared to Ac_4_GlcNAz, however, no evidence exists in literature that the corresponding monosaccharide (ManNAc) is incorporated in Arabidopsis glycans. In addition, no evidence exist that mannosamine can be used as a precursor for the biosynthesis of other sugar derivatives in plants [34]. Indeed, Arabidopsis seedlings incubated with Ac_4_ManNAz showed no labeling. This confirms that Ac_4_ManNAz is indeed not present in Arabidopsis cell walls and supports the experiments described above that indicated that Ac_4_GlcNAz, Ac_4_FucAz and Ac_3_ArabAz incorporation is mediated by active metabolism.

### Azido-monosaccharide incorporation is time-dependent and mediated by passive or active transport

To investigate if active cellular metabolism is necessary for incorporation of azido-monosaccharides, whole seedlings were killed and fixated by 4 % paraformaldehyde. These fixated seedlings could then be used to distinguish between two scenarios, one where incorporation takes place via an active glycan salvage pathway [12], or alternatively, a scenario where azido-monosaccharides are passively absorbed onto external cell walls. Fixation resulted in slightly more background fluorescence, but the intensity of fixated seedlings incubated with Ac_4_GlcNAz was equal to fixated DMSO control seedlings (Additional file 6). This suggests that Ac_4_GlcNAz is actively incorporated through the plant cell metabolism rather than through non-specific absorption of the azido-monosaccharide to the plant cell wall. Similar results have been reported for alkyne-monosaccharides and a different azido-monosaccharide in Arabidopsis [18, 19, 22].

**Fig. 4:**
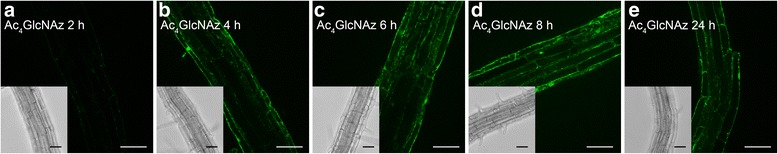
Optical sections of 4 day old Arabidopsis seedling roots incubated for 2 (**a**), 4 (**b**), 6 (**c**), 8 (**d**) and 24 h (**e**) with 25 μM Ac_4_GlcNAz, followed by labeling through a copper-catalyzed click reaction with Alexa Fluor® 488 alkyne. Scale bars = 50 μm (see Additional file 14 for high resolution)

Next, the optimal incubation time of Arabidopsis seedlings in MS with 25 μM Ac_4_GlcNAz was determined (Fig. 4 & Additional file 7). Visible incorporation (Fig. 4b) was observed after 4 h of incubation with 25 μM Ac_4_GlcNAz, while no incorporation was observed after 2 h (Fig. 4a). The brightest fluorescence was observed after 6 and 8 h of incubation (Fig. 4c–d & Additional file 7). This supports the idea that an active glycan salvage pathway is required for incorporation of Ac_4_GlcNAz. In a scenario involving passive adsorption weak fluorescence would already be expected after 2 h of incubation. Fluorescence decreased after 24 h incubation, which is most likely due to spreading of Ac_4_GlcNAz through the whole Arabidopsis root or an increased competition with natural *N*-acetyl-d-glucosamine synthesized by the plant itself. The time-dependent incorporation was also investigated for Ac_4_FucAz and Ac_3_ArabAz. In contrast to Ac_4_GlcNAz, incorporation of Ac_4_FucAz and Ac_3_ArabAz was visible after 2 h, but the best incorporation was observed after 24 h (Additional files 8 and 9).

**Fig. 5:**
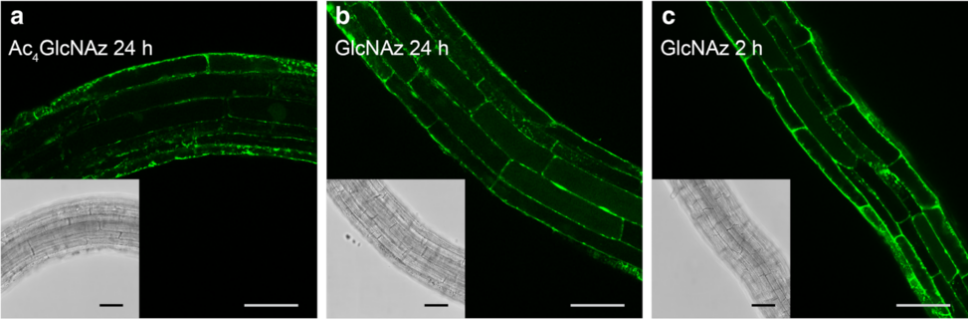
Optical sections of 4 day old Arabidopsis seedling roots incubated for 24 h with 25 μM acetylated Ac_4_GlcNAz (**a**) or non-acetylated GlcNAz (**b**), followed by labeling through a copper-catalyzed click reaction with Alexa Fluor® 488 alkyne. Early incorporation with GlcNAz was observed with seedlings incubated for 2 h with 25 μM GlcNAz (**c**). Scale bars = 50 μm (see Additional file 14 for high resolution)

It is generally believed that the more hydrophobic acetylated monosaccharide probes, compared to their more polar non-acetylated version, end up inside plant cells via passive uptake [17–19]. Roberts et al. reported that root tissues of higher plants rapidly take up d-Glucosamine from aqueous medium for incorporation into root tissue [35]. They also observed active uptake of *N*-acetyl-d-glucosamine, albeit 10 times slower, via the same pathway [35]. Since this indicated that *N*-acetylglucosamine – not acetylated at any of the hydroxyl groups – is actively taken up by the roots of Arabidopsis, we wondered whether the corresponding non-acetylated GlcNAz could also be incorporated similar to the fully acetylated analog, Ac_4_GlcNAz. To investigate this, Arabidopsis seedlings were incubated for 24 h with either 25 μM Ac_4_GlcNAz or 25 μM GlcNAz (Fig. 5a and b). Incorporation was visible for both Ac_4_GlcNAz and GlcNAz with an almost similar fluorescent strength. This can indicate a maximum uptake for both sugar analogues after 24 h. It also suggests that GlcNAz is actively taken up via a cell membrane transport system as its polarity makes passing the fatty non-polar cell membranes via passive transport implausible. Non-acetylated GlcNAz uptake and incorporation was already visible after 2 h (Fig. 5c), this is in line with previous observations [35], although GlcNAz is not directly comparable with *N-*acetyl-d-glucosamine. To determine the location of Ac_4_GlcNAz and GlcNAz incorporation the seedlings were stained with propidium iodide (PI) after the copper-catalyzed click reaction. Overlap of both incorporated monosaccharides with PI was observed, indicating cell wall labeling (Additional file 10), and no differences in labeling pattern between Ac_4_GlcNAz and GlcNAz were observed. Taken together, this indicates that Arabidopsis is capable of active uptake of GlcNAz and it is probably salvaged and incorporated via the same pathway as Ac_4_GlcNAz.

### Incorporation of Ac_4_GalNAz indicates GalNAc is metabolised in *Arabidopsis*

**Fig. 6:**
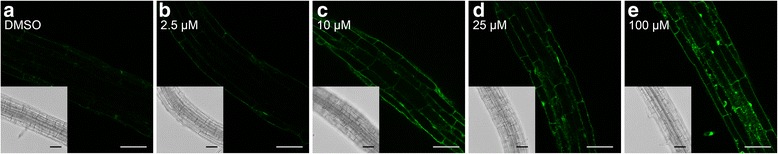
Optical sections of 4 day old Arabidopsis seedling roots incubated for 48 h with 2.5 μM (**b**), 10 μM (**c**), 25 μM (**d**), and 100 μM (**e**) Ac_4_GalNAz, followed by labeling through a copper-catalyzed click reaction with Alexa Fluor® 488 alkyne. As a control, seedlings were treated with 0.01 % DMSO (**a**). Scale bars = 50 μm (see Additional file 14 for high resolution)

*N*-Acetylgalactosamine (GalNAc) is not documented to be present in Arabidopsis glycans [12], while GalNAc is found in several other higher plants [36, 37] and in *N-*glycans of algae [38]. Glycosylation with GalNAc in Arabidopsis has only been documented in genetically engineered plant cell systems of this plant species [39]. Still, while it is not known whether GalNAc is incorporated into glycans, there is evidence for a UDP-GlcNAc nucleotidyltransferase in Arabidopsis that is capable of converting GalNAc-1-P into its corresponding UDP-GalNAc [40]. In addition, a transporter was recently discovered in Arabidopsis that is capable of transporting both UDP-GlcNAc and UDP-GalNAc [21]. The presence of both an UDP-GalNAc transporter and the GalNAc-compatible transferase indicates that GalNAc might be salvaged or metabolized by Arabidopsis. For this reason, we investigated if this glycan metabolism could potentially be studied with a GalNAc-derived azido-monosaccharide, *N*-azidoacetyl-galactosamine (Ac_4_GalNAz). Ac_4_GalNAz was prepared according to a procedure described by Bertozzi et al. [26] and then co-incubated with Arabidopsis seedlings for 24 h using different concentrations (2.5–100 μM; Additional file 11). An incorporation signal for Ac_4_GalNAz was observed after 24 h (Additional file 11). However, the incubation time was prolonged because we observed lower fluorescence compared to the other azido-monosaccharides that we studied using the same incubation time. Increasing the incubation time to 48 h indeed improved the incorporation (Fig. 6).

This might indicate that salvage and incorporation of Ac_4_GalNAz - compared to the monosaccharide analogs known to be present in Arabidopsis glycans - takes place via a lengthier pathway. The pathway may include an unknown epimerase that converts *N*-acetylgalactosamine, or a derivative thereof, to the corresponding *N*-acetylglucosamine epimer. An epimerase has been discovered in barley that reversibly interconverts UDP-GalNAc and UDP-GlcNAc and of which a homolog exist in Arabidopsis [41]. Maximum incorporation with 25 μM Ac_4_GalNAz was observed in a time-frame of 24 h (Additional file 11d), whereas 100 μM Ac_4_GalNAz was required (Fig. 6e) to reach saturation with an incubation time of 48 h. The relative high concentration required after 48 h, is consistent with the other lengthier time incubation experiments with azido-monosaccharides.

### Copper-free click reactions are good alternatives to label glycans in Arabidopsis roots

**Fig. 7:**
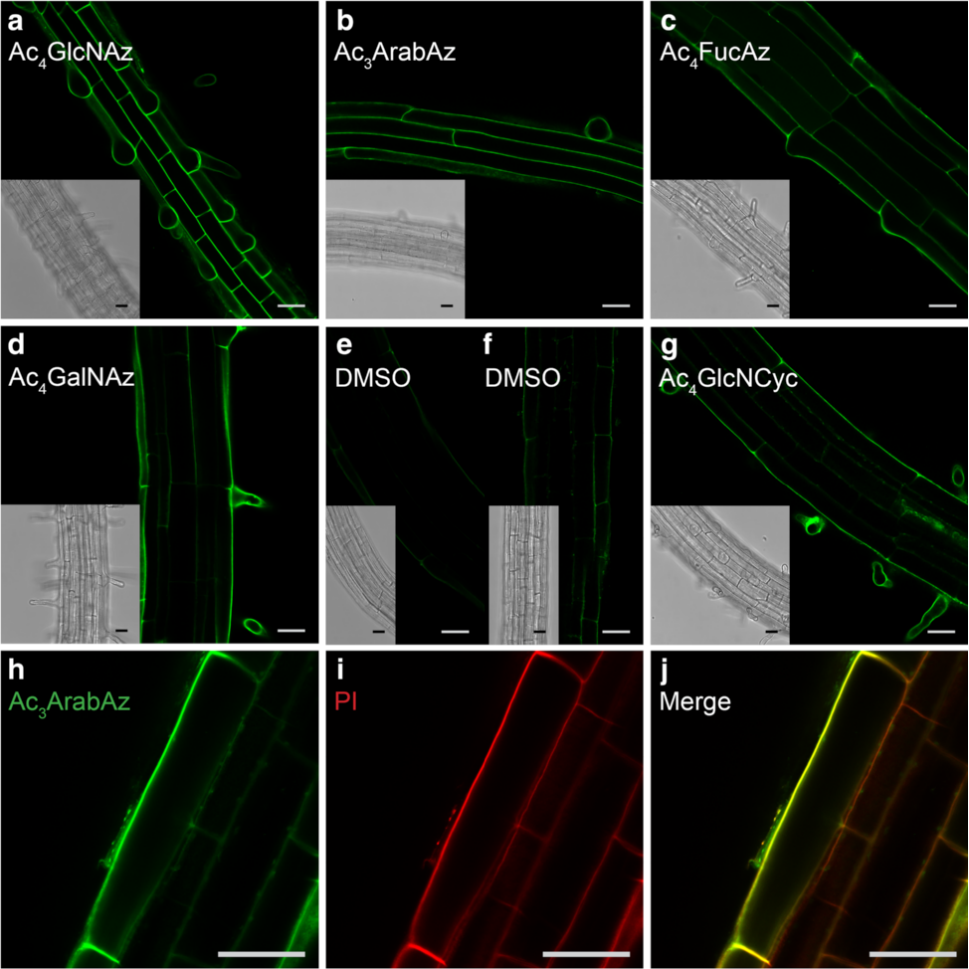
Optical sections of 4 day old Arabidopsis seedling roots incubated for 24 h with 25 μM acetylated Ac_4_GlcNAz (**a**), 100 μM Ac_3_ArabAz (**b**), 25 μM Ac_4_FucAz (**c**), 25 μM Ac_4_GalNAz (**d**) or 50 μM GlcNCyc (**g**) followed by labeling through strain-promoted alkyne-azide cycloaddition with DBCO-PEG4-ATTO-488 (**a**–**d**) or an inverse electron demand Diels-Alder click reaction with Tetrazine-ATTO-488 (**g**). As a control, seedlings were treated with 0.01 % DMSO followed by labeling through strain-promoted alkyne-azide cycloaddition with DBCO-PEG4-ATTO-488 (**e**) or an inverse electron demand Diels-Alder click reaction with Tetrazine-ATTO-488 (**f**). Seedling roots treated with DBCO-PEG4-ATTO-488 labeled Ac_3_ArabAz (100 μM, 24 h) (**f**, **j**) counterstained Propidium Iodide (PI, 0.05 %) to visualize cell walls (**i**, **j**). Yellow color indicates overlap of the two dyes (**j**). Scale bars = 25 μm (see Additional file 14 for high resolution)

A drawback of the studies reported until now that use monosaccharide probes to image plant glycans is that they all use a copper-catalyzed click reaction to attach the fluorescent reporter group to the metabolically incorporated glycans. The copper required to catalyze this reaction is known to be toxic to Arabidopsis and therefore might influence the outcome of the labeling and imaging experiments in which it is used. This side effect is indeed also observed by us in the slightly inhomogeneous labeling after the copper-catalyzed click reaction, which damages the cell wall and in a few instances also caused minor internal labeling. The toxic effect that copper has on Arabidopsis seedlings was also observed by Anderson and coworkers [18, 22], who applied copper-catalyzed reactions to label alkyne-monosaccharides. To circumvent the use of copper ions, an alternative copper-free click reaction, the so-called strain-promoted alkyne-azide cycloaddition (SPAAC) has been developed, which is bio-orthogonal and can be applied to living cells [42]. It has not been applied towards azido-monosaccharide analog probes in Arabidopsis so far. This reaction is still rapid enough for biological applications, for instance when an azide-containing probe is reacted with an aliphatic cyclooctyne (BCN) [43] or dibenzocyclooctyne (DBCO) [44, 45]. Labeling of azido-monosaccharides *via* SPAAC has an advantage compared to the copper-catalyzed click reaction, since it does not damage living cells. However, while the alkyne-monosaccharides reported earlier cannot utilize SPAAC, our azido-monosaccharide probes do have the potential to be labeled through this click reaction. To investigate copper-free labeling of plant glycans via SPAAC, seedlings were labeled after incubation with Ac_4_GlcNAz (25 μM, 24 h), Ac_4_FucAz (25 μM, 24 h), Ac_3_ArabAz (100 μM, 24 h) or Ac_4_GalNAz (25 μM, 24 h) with a solution containing 1 μM DBCO-PEG4-ATTO-488 in MS for 1 h. The resulting metabolically-labeled seedlings showed bright fluorescence and low background (Fig. 7a–d). In contrast, seedlings incubated with 0.01 % DMSO showed only background fluorescence (Fig. 7e).

Comparison of seedlings labeled with Ac_4_GlcNAz and Ac_3_ArabAz with propidium iodide-labeled seedlings showed excellent overlap, indicating incorporation of the azido-monosaccharides in the cell wall glycans (Fig. 7h–j and Additional file 12). Experiments with a more apolar DBCO-fluorophore without a PEG-spacer and a BCN-derived fluorophore were not successful and extensive non-specific absorption of the fluorophore (also as micelles) to the cell wall was observed.

In addition to SPAAC, other bio-orthogonal copper-free click reactions are also known. The inverse electron demand Diels-Alder (invDA) reaction between tetrazines and strained alkenes/alkynes has gained popularity as a very fast and bio-orthogonal complementary reaction to SPAAC [46]. We investigated whether this reaction could also be used for labeling plant glycans. Tetrazines conjugated to a fluorescent reporter group are typically used for labeling and we choose the smallest possible tetrazine reaction partner, a methyl-cyclopropene, as a chemical handle on an *N*-acetylglucosamine derivative. Known GlcNAc derivative with a methyl-cyclopropene (GlcNCyc), was prepared via an adapted procedure of Prescher [47] and Wittmann and co-workers [48]. Arabidopsis seedlings incubated with GlcNCyc for 24 h showed bright fluorescence, when clicked with 15 μM Tetrazine-ATTO-488 (Fig. 7g), while a DMSO control did not show appreciable fluorescence (Fig. 7f). Besides, compared to the other clickable dyes used in this study, it was observed that Alexa® Fluor-tetrazine was less prone to stick to the cell wall and more water soluble than alkyne and DBCO dyes. This has the advantage that the fluorophore can be used at higher concentrations. These preliminary experiments with the SPAAC and invDA copper-free click reactions resulted in a more uniform staining. In addition, these mild labeling reactions do not require cytotoxic copper, which enables experiments that go beyond snapshot images of plant seedlings.

## Conclusions

In this study, the toolbox of clickable monosaccharide analogs for glycan labeling in Arabidopsis seedlings has been expanded to allow the incorporation and direct visualization of five relevant plant monosaccharide analogs in complex cell wall-bound glycans. The clickable glycan analogs Ac_4_GlcNAz, Ac_3_ArabAz, Ac_4_FucAz and Ac_4_GalNAz were successfully metabolically incorporated and visualized in glycans of Arabidopsis seedling roots. The novel Ac_3_ArabAz for the first time allows for direct imaging of l-arabinose, one of the most common plant *O-*glycans and an important constituent of plant cell wall polysaccharides [27–30]. The incorporation of Ac_4_GalNAz we observe supports the possibility of an epimerase in Arabidopsis that converts GalNAz to GlcNAz. During the preparation of this manuscript Chen and coworkers reported on the metabolic incorporation and imaging of *N*-linked glycans in Arabidopsis with Ac_4_GlcNAz [24]. Our here reported results with this azido-monosaccharide are in correspondence with their work, and provide additional details on Ac_4_GlcNAz metabolic incorporation and imaging through the glycan salvage pathway. For example, we show that Ac_4_GlcNAz is already being incorporated after 4 h and that GlcNAz (non-acetylated) can also be salvaged, probably via active transport, within 2 h. Finally, earlier reports on the metabolic incorporation and imaging of monosaccharide analogs, including Ac_4_GlcNAz, rely solely on labeling through copper-catalyzed click chemistry. Although copper-catalyzed click reactions often work well, the toxicity of copper here led to damage of the cell wall, emphasizing the need for copper-free clickable analogs for long-term or spatiotemporal experiments. We here show for the first time that the strain-promoted azide-alkyne cycloaddition (SPAAC) and inverse electron demand Diels-Alder (invDA) click reactions allow for improved imaging of metabolic labeling with our azido-monosaccharides and a cyclopropene-GlcNAc derivative. The application of these improved copper free labeling methods can be generalized and extended to already existing and future click chemistry-enabled monosaccharide analogs in Arabidopsis. Taken together with the fact that the SPAAC and invDA reactions are bio-orthogonal *and* orthogonal with respect to each other, this will allow for *in vivo* and dual plant glycan labeling applications. Overall our results here and other recently published studies [18, 19, 24] promise a bright future for the Metabolic Oligosaccharide Engineering (MOE) methodology to enable the direct spatiotemporal imaging of complex glycans in living plants [16].

## Methods

### Growth of *Arabidopsis Thaliana*

Wild type *Arabidopsis thaliana* (Col-0) seeds were surface sterilized in a mixture of commercial bleach and ethanol (v/v; 1/4) for 15 min followed by washing with ethanol (2 times) and drying. First a cold shock was applied on all sterilized seeds by placing them in a fridge (5 °C) for at least 2 days with a maximum of one week while on filter paper, pre-wetted with 2 mL Milli-Q water, in a petri dish. Seeds were grown on half Murashige and Skoog medium (MS) [49] with vitamins in a petri dish (0.8 % plant agar) in a climate room on the shelf lit by Philips 36 W/840 lamps (120 μmol/m^2^ s) under long-day conditions (16 h light/8 h dark) at 22 °C. Young seedlings of 4 or 5 days old were used for incubation experiments.

### Incubation of Arabidopsis

Five young seedlings were put together in single well of a 24-well plate containing click-compatible azido-monosaccharide in half MS. After incubation time, 5 wells were filled with 2 mL half MS medium containing 0.05 % Tween 20. Plants were dipped in each well for 15 s to wash away the excess of azido-monosaccharide. The seedlings were directly transferred to a new 24-well plate for labeling through either a 1) copper-catalyzed click-labeling 2) a SPAAC labeling or 3) a Diels alder-cycloaddition labeling.

### Copper-catalyzed click-labeling

Click-iT cell reaction kit (supplier: Invitrogen) was used for all copper-catalyzed “click” reactions. The labeling was carried out according to the procedure in the manual of Invitrogen except for the reaction time that was prolonged to 45 min. For the Alexa-fluor 488 fluorophore a concentration of 0.1 μM was found to be the most optimal. The excess of fluorophore was removed by washing the seedlings 4× in 2 mL half MS containing 0.05 % Tween 20. Duration of the sequential washings steps were respectfully 5, 10, 5 and 10 min. After washing the seedlings were stored for with a maximum time of 2 h in half MS (not containing Tween 20) before visualization by confocal microscopy.

### SPAAC labeling

SPAAC reactions were performed in 2 mL of 1 μM DBCO-PEG4-ATTO-488 in half MS medium. Reaction time was 1 h. Washing and storage was similar to the copper-catalyzed click reaction described above. The washing times were prolonged to 4 × 10 min.

### Diels-Alder cycloadditions

Reactions were performed in 1 mL of 15 μM Tetrazine-ATTO-488 in half MS medium. All other procedures were similar to the SPAAC reactions described above.

### Seedling fixation with paraformaldehyde

As a negative control, seedlings were fixated in 4 % paraformaldehyde solution in PBS (commercially available). Five seedlings were put together in 2 mL of the paraformaldehyde solution for 30 min. Afterwards seedlings were washed two times in 2 mL 0.5 MS before incubation with click-compatible azido-monosaccharides as discussed before.

### Toxicity test

Toxicity tests were performed based on growth of the plant. Agar plates containing the described azido-monosaccharides analogs were used for the growth experiments with young *Arabidopsis* seedlings for 8 days. Azido-monosaccharides were added after sterilization of the medium, when it was cooled down to approximately 60 °C and before pouring the medium in a petri dish. *Arabidopsis* seedlings were subsequently germinated and grown on agar plates containing the different azido-monosaccharide solutions in ½ MS with 0.8 % plant agar. After 8 days of growth, the white part of the root was measured from leaves till root tip.

### Microscopy and image analysis

Roots of seedlings were imaged with a Leica TCS SP8 confocal microscope (488 nm laser excitation, 534-571 emission filter and 600-650 emission filter for PI) using a 40X water immersion objective. Image J was used to process images. All images within the same experiment were adjusted to the same color balance. Mean fluorescence was calculated in Image J (rsbweb.nih.gov/ij) using freehand tool to select the cell boundary of epidermal cells and to measure the mean pixel intensity. The standard deviation was determined based on the difference in the fluorescence intensity throughout the cells of a seedling. Data of those cells were collected from 3–4 seedlings per treatment and imaged using identical exposure settings.

### General information and methods for synthesis

Ac_4_GlcNAz, Ac_4_GalNAz and GlcNCyc were prepared according to literature procedures [26]. GlcNCyc was prepared according to a literature procedure by Presher [47] and Wittmann et al. [48], while the synthesis of one of the intermediates in this synthesis – the cyclopropane tag – has been adapted and described in Additional file 13. The synthesis of Ac_4_FucAz, Ac_3_ArabAz are described in Additional file 13.

## Additional files

Additional file 1: Evaluation of toxicity of azido-monosaccharides. (DOCX 22 kb)
Additional file 2: Concentration-dependent Ac_4_GlcNAz incorporation. (DOCX 207 kb)
Additional file 3: Concentration-dependent Ac_4_FucAz incorporation. (DOCX 338 kb)
Additional file 4: Concentration-dependent Ac_3_ArabAz incorporation. (DOCX 351 kb)
Additional file 5: Optical sections of the control experiments of Ac_4_ManNAz incorporation. (DOCX 338 kb)
Additional file 6: Control experiments with paraformaldehyde fixed seedlings. (DOCX 319 kb)
Additional file 7: Quantified time-dependent GlcNAz incorporation. (DOCX 25 kb)
Additional file 8: Time-course of Ac_4_FucAz incorporation in elongating root cells. (DOCX 287 kb)
Additional file 9: Time-course of Ac_3_ArabAz incorporation in elongating root cells. (DOCX 285 kb)
Additional file 10: Comparison of non-acetylated Ac_4_GlcNAz labeled seedlings with PI stain. (DOCX 775 kb)
Additional file 11: Concentration-dependent Ac_4_GalNAz incorporation (24 h incubation). (DOCX 322 kb)
Additional file 12: Comparison of Ac_4_GlcNAz and Ac_3_ArabAz labeled seedlings with PI stain. (DOCX 1277 kb)
Additional file 13: Synthesis procedures and characterization data for azido-monosaccharides. (DOCX 1338 kb)
Additional file 14: Figures in high resolution. (ZIP 22425 kb)

## Abbreviations

Ac_3_ArabAz: 1,2,3, Tri-*O*-acetyl-5-azido-5-deoxy*-* l-arabinofuranose
Ac_4_FucAz: 1,2,3,4-tetra-*O*-acetyl-6-azido-l *-*fucose
Ac_4_GalNAz: 1,3,4,4-tetra-*O*-acetyl-N-azidoacetyl-α,β-d-galactosamine
Ac_4_GlcNAz: 1,3,4,4-tetra-*O*-acetyl-N-azidoacetyl-α,β-d-glucosamine
Ac_4_ManNAz: 1,3,4,4-tetra-*O*-acetyl-*N*-azidoacetyl-α,β-d-mannosamine
DBCO: Dibenzocyclooctyne
DMSO: Dimethyl sulfoxide
GalNAc: *N*-acetyl-d-galactosamine
GlcNAz: *N*-azidoacetylglucosamine
GlcNCyc: 1,3,4,4-tetra-*O*-acetyl-*N*-methylcyclopropene-α,β-d-glucosamine
PI: Propidium iodide
SPAAC: Strain promoted azido-alkyne click chemistry
