# Supplementary material for: Direct imaging of glycans in Arabidopsis roots via click labeling of metabolically incorporated azido-monosaccharides
Source: BMC Plant Biol. 2016 Oct 10;16:220. doi: 10.1186/s12870-016-0907-0 (PMC5056477; doi:10.1186/s12870-016-0907-0)
Supplement: Additional file 1: — Evaluation of toxicity of azido-monosaccharides. (DOCX 22 kb) [file 12870_2016_907_MOESM1_ESM.docx]

Additional file 1. Arabidopsis growth on agar plate with various azido-monosaccharides. *Arabidopsis* seedlings were germinated and grown on agar plates containing the different azido-monosaccharide solutions in ½ MS with 0.8% plant agar. After 8 days of growth, the white part of the root was measured from leaves till root tip. Root length measured after 8 days of growth. Concentrations (10, 25 or 100) are in µM). Error bars represent standard deviations (n= 20 seedlings for each experiment, n=60 for control experiments). No significant difference was observed (one-way anova test, P<0.05).
