# Supplementary material for: Direct imaging of glycans in Arabidopsis roots via click labeling of metabolically incorporated azido-monosaccharides
Source: BMC Plant Biol. 2016 Oct 10;16:220. doi: 10.1186/s12870-016-0907-0 (PMC5056477; doi:10.1186/s12870-016-0907-0)
Supplement: Additional file 2: — Concentration-dependent Ac4GlcNAz incorporation. (DOCX 207 kb) [file 12870_2016_907_MOESM2_ESM.docx]

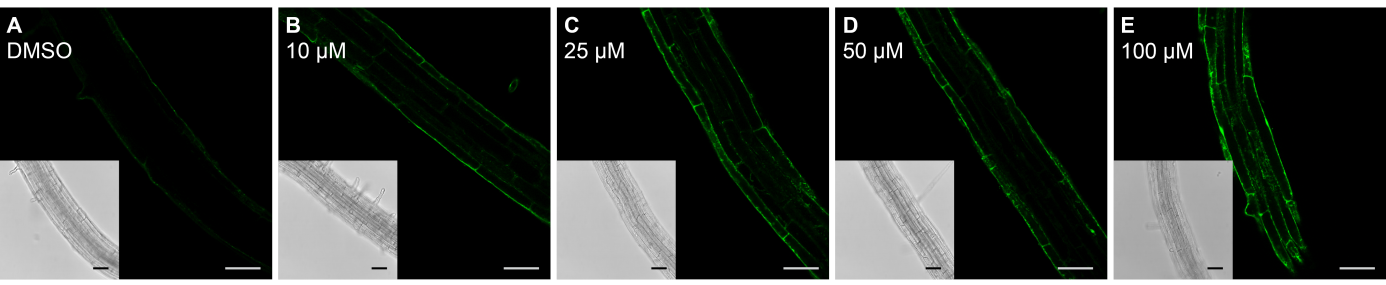


Additional File 2A. Optical sections of 4 day old Arabidopsis seedling roots incubated for 24 hours with 10 µM (b), 25 µM (c), 50 µM (d), and 100 µM (e) GlcNAz, followed by labelling through a copper-catalysed click-reaction with Alexa Fluor® 488 alkyne. As a control, seedlings were treated with 0.01 % DMSO (a). Scale bars = 50 μm.


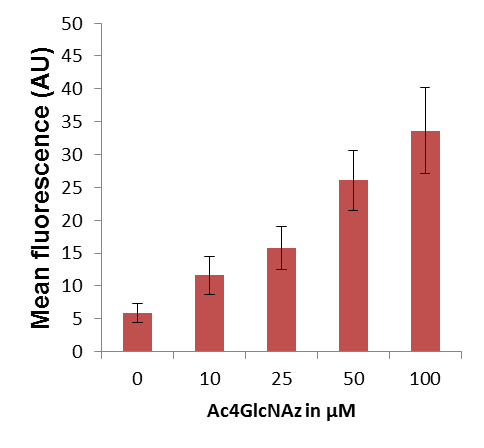


Additional File 2B. Mean fluorescence intensity of the epidermal cells of 4 day old Arabidopsis seedling roots incubated for 24 hours with 0-100 μM Ac_4_GlcNAz. The error bars represent the S.D. in the fluorescent intensity throughout the cells of seedlings . Data of those cells were collected from 3-4 seedlings per treatment.
