# Supplementary material for: Direct imaging of glycans in Arabidopsis roots via click labeling of metabolically incorporated azido-monosaccharides
Source: BMC Plant Biol. 2016 Oct 10;16:220. doi: 10.1186/s12870-016-0907-0 (PMC5056477; doi:10.1186/s12870-016-0907-0)
Supplement: Additional file 3: — Concentration-dependent Ac4FucAz incorporation. (DOCX 338 kb) [file 12870_2016_907_MOESM3_ESM.docx]

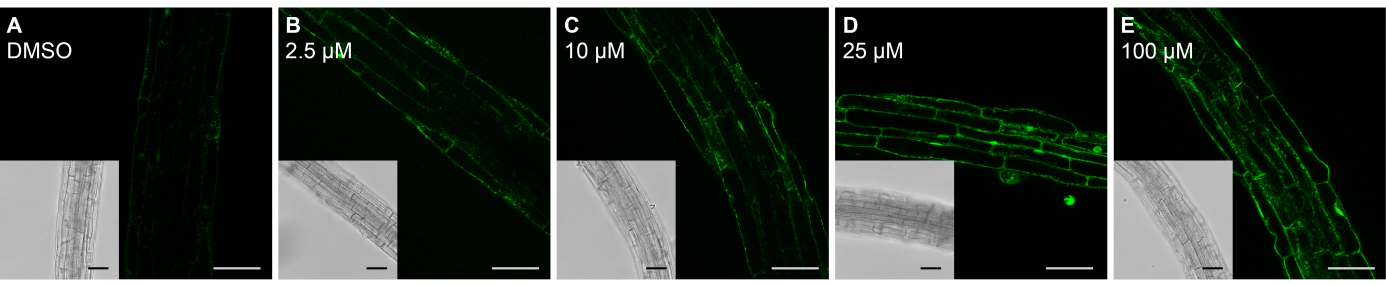


Additional File 3. Optical sections of 4 day old Arabidopsis seedling roots incubated for 24 hours with 2.5 µM (b), 10 µM (c), 25 µM (d), and 100 µM (e) FucAz, followed by labelling through a copper-catalysed click-reaction with Alexa Fluor® 488 alkyne. As a control, seedlings were treated with 0.01 % DMSO (a). Scale bars = 50 μm.
