# Supplementary material for: Direct imaging of glycans in Arabidopsis roots via click labeling of metabolically incorporated azido-monosaccharides
Source: BMC Plant Biol. 2016 Oct 10;16:220. doi: 10.1186/s12870-016-0907-0 (PMC5056477; doi:10.1186/s12870-016-0907-0)
Supplement: Additional file 5: — Optical sections of the control experiments of Ac4ManNAz incorporation. (DOCX 338 kb) [file 12870_2016_907_MOESM5_ESM.docx]

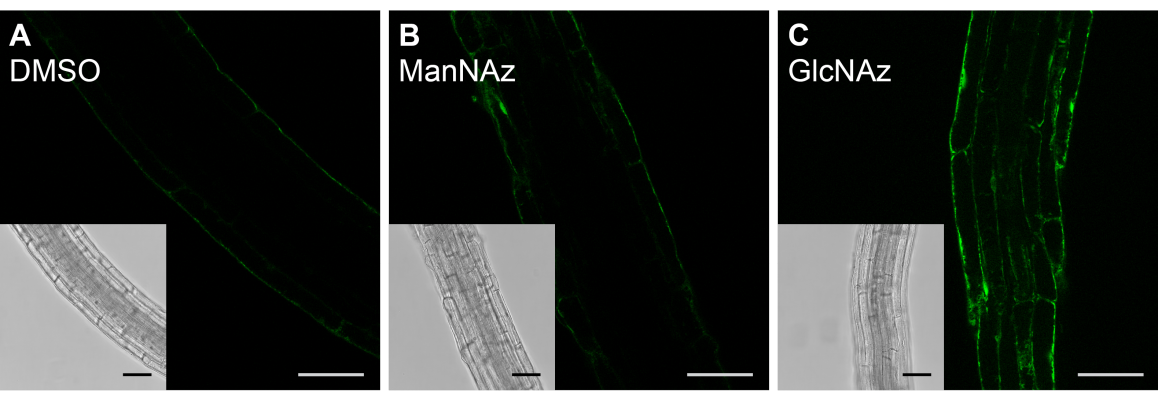


Additional File 5. DMSO-incubated control for the corresponding ManNAz experiment. Optical sections of 4 day old Arabidopsis seedling roots incubated for 24 hours with 0.01 % DMSO as a control (a), 25 µM ManNAz (b), or 25 µM GlcNAz (c), followed by labelling through a copper-catalysed click-reaction with Alexa Fluor® 488 alkyne. Scale bars = 50 μm.
