# Supplementary material for: Direct imaging of glycans in Arabidopsis roots via click labeling of metabolically incorporated azido-monosaccharides
Source: BMC Plant Biol. 2016 Oct 10;16:220. doi: 10.1186/s12870-016-0907-0 (PMC5056477; doi:10.1186/s12870-016-0907-0)
Supplement: Additional file 6: — Control experiments with paraformaldehyde fixed seedlings. (DOCX 319 kb) [file 12870_2016_907_MOESM6_ESM.docx]

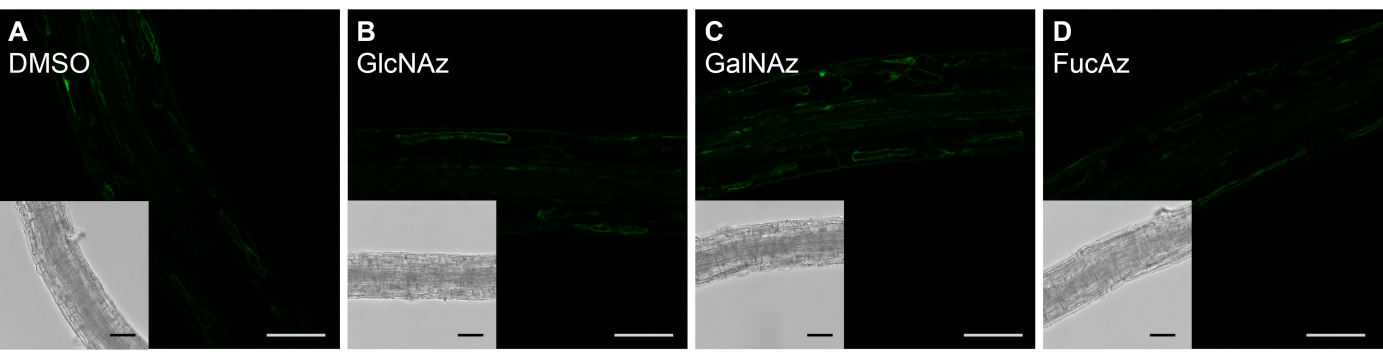
 Additional File 6. Azido-monosaccharide incorporation requires cell viability. Optical sections of 4 day old Arabidopsis seedling roots fixated in 4% paraformaldehyde solution in PBS incubated for 24 hours with 25 µM GlcNAz (b), 25 µM GalNAz (c), 25 µM FucAz (d), followed by labelling through a copper-catalysed click-reaction with Alexa Fluor® 488 alkyne. As a control, seedlings were treated with 0.01 % DMSO (a). Scale bars = 50 μm.
