# Supplementary material for: Direct imaging of glycans in Arabidopsis roots via click labeling of metabolically incorporated azido-monosaccharides
Source: BMC Plant Biol. 2016 Oct 10;16:220. doi: 10.1186/s12870-016-0907-0 (PMC5056477; doi:10.1186/s12870-016-0907-0)
Supplement: Additional file 7: — Quantified time-dependent GlcNAz incorporation. (DOCX 25 kb) [file 12870_2016_907_MOESM7_ESM.docx]

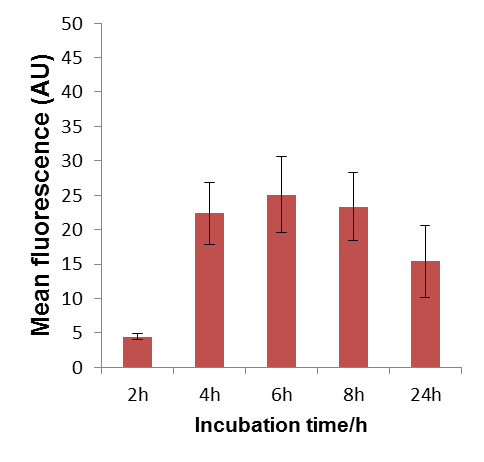


Additional File 7. Mean fluorescence intensity of the epidermal cells of 4 day old Arabidopsis seedling roots illustrating the time-dependent uptake of 25 μM Ac_4_GlcNAz. The error bars represent the S.D. in the fluorescent intensity throughout the cells of seedlings . Data of those cells were collected from 3-4 seedlings per treatment.
