# Supplementary material for: Direct imaging of glycans in Arabidopsis roots via click labeling of metabolically incorporated azido-monosaccharides
Source: BMC Plant Biol. 2016 Oct 10;16:220. doi: 10.1186/s12870-016-0907-0 (PMC5056477; doi:10.1186/s12870-016-0907-0)
Supplement: Additional file 8: — Time-course of Ac4FucAz incorporation in elongating root cells. (DOCX 287 kb) [file 12870_2016_907_MOESM8_ESM.docx]

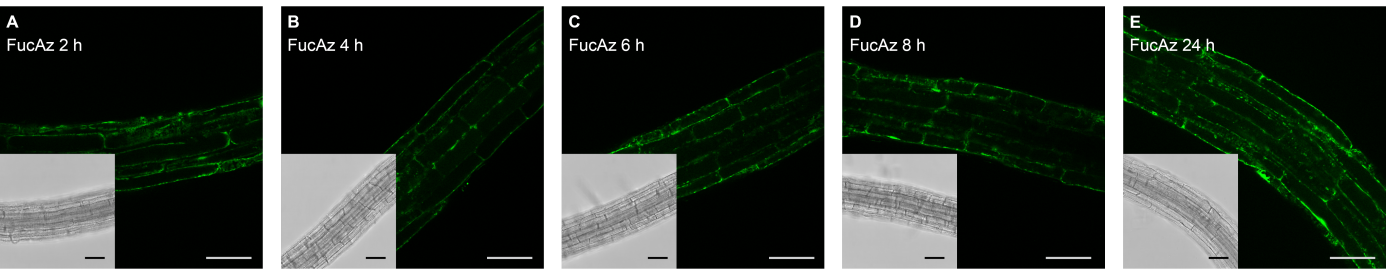
Additional File 8A. Optical sections of 4 day old Arabidopsis seedling roots incubated for 2 (a), 4 (b), 6 (c), 8 (d) and 24 hours (e) with 25 µM Ac_4_FucAz, followed by labelling through a copper-catalysed click-reaction with Alexa Fluor® 488 alkyne. Scale bars = 50 μm.

Additional File 8B. Mean fluorescence intensity of the epidermal cells of 4 day old Arabidopsis seedling roots illustrating the time-dependent uptake of 25 μM Ac_4_FucAz. The error bars represent the S.D. in the fluorescent intensity throughout the cells of seedlings. Data of those cells were collected from 3-4 seedlings per treatment.
