# Supplementary material for: Direct imaging of glycans in Arabidopsis roots via click labeling of metabolically incorporated azido-monosaccharides
Source: BMC Plant Biol. 2016 Oct 10;16:220. doi: 10.1186/s12870-016-0907-0 (PMC5056477; doi:10.1186/s12870-016-0907-0)
Supplement: Additional file 10: — Comparison of non-acetylated Ac4GlcNAz labeled seedlings with PI stain. (DOCX 775 kb) [file 12870_2016_907_MOESM10_ESM.docx]

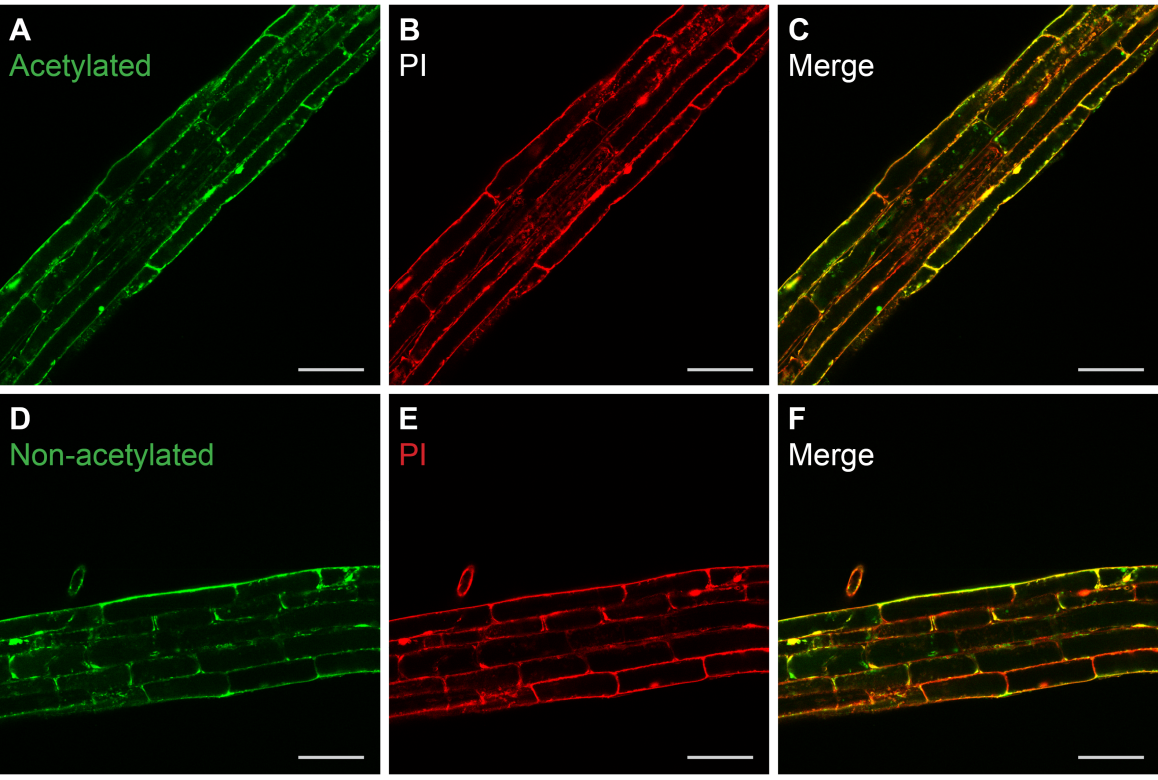


Additional File 10A. Optical sections of 4 day old Arabidopsis seedling roots incubated for 24 hours with 25 µM GlcNAz (a-c) or 25 µM non-acetylated GlcNAz (d-f), followed by labelling through a copper-catalysed click-reaction with Alexa Fluor® 488 alkyne. Seedling roots treated with Alexa Fluor® 488 alkyne labelled GlcNAz (25 µM, 24 h) (a,c) were counterstained with Propidium Iodide (PI, 0.05%) to visualize cell walls (b,c). Similarly, seedling roots treated with Alexa Fluor® 488 alkyne labelled non-acetylated GlcNAz (25 µM, 24 h) (d,f) were counterstained with Propidium Iodide (PI, 0.05%) to visualize cell walls (e,f). Yellow colour indicates overlap of the two dyes (c,f). Scale bars = 50 μm.


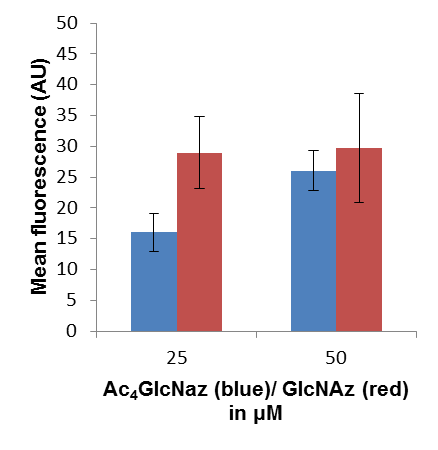


Additional File 10B. Mean fluorescence intensity of the epidermal cells of 4 day old Arabidopsis seedling roots incubated for 24 hours with 25-50 acetylated Ac_4_GlcNAz or unprotected GlcNAz. The error bars represent the S.D. in the fluorescent intensity throughout the cells of seedlings . Data of those cells were collected from 3-4 seedlings per treatment.
