# Supplementary material for: Direct imaging of glycans in Arabidopsis roots via click labeling of metabolically incorporated azido-monosaccharides
Source: BMC Plant Biol. 2016 Oct 10;16:220. doi: 10.1186/s12870-016-0907-0 (PMC5056477; doi:10.1186/s12870-016-0907-0)
Supplement: Additional file 12: — Comparison of Ac4GlcNAz and Ac3ArabAz labeled seedlings with PI stain. (DOCX 1277 kb) [file 12870_2016_907_MOESM12_ESM.docx]

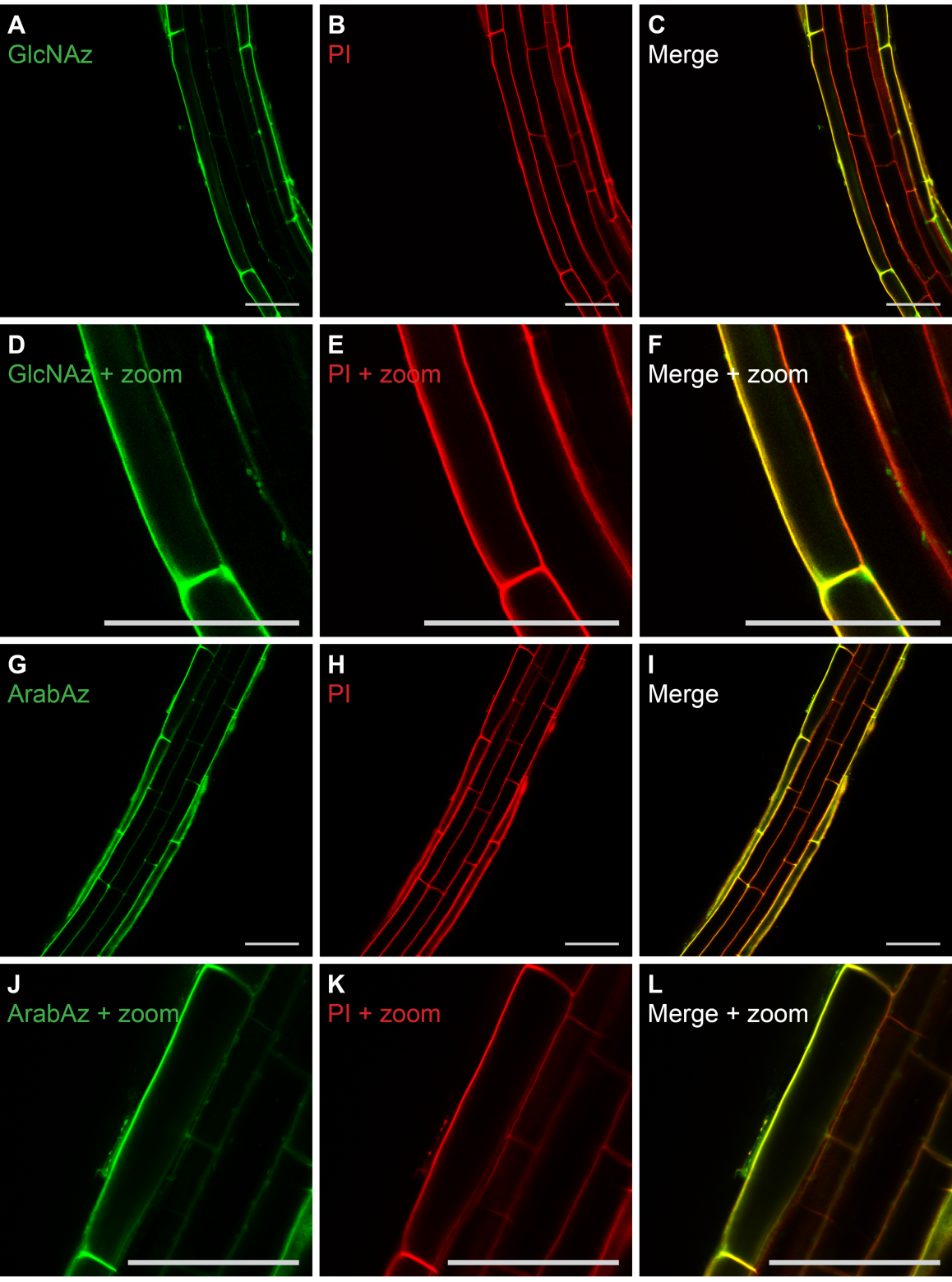


Additional File 12. Optical sections of 4 day old Arabidopsis seedling roots incubated for 24 hours with 25 µM GlcNAz (a-f) or 100 µM ArabAz (g-l), followed by labelling through strain-promoted alkyne-azide cycloaddition with DBCO-PEG4-ATTO-488. Seedling roots treated with DBCO-PEG4-ATTO-488 labelled GlcNAz (25 µM, 24 h) (a,c,d,f) were counterstained with Propidium Iodide (PI, 0.05%) to visualize cell walls (b,c,e,f). Similarly, Seedling roots treated with DBCO-PEG4-ATTO-488 labelled ArabAz (100 µM, 24 h) (g,i,j,l) were counterstained with Propidium Iodide (PI, 0.05%) to visualize cell walls (h,i,k,l). Yellow colour indicates overlap of the two dyes (c,f,i,l). Scale bars = 50 μm.
