# Supplementary material for: Direct imaging of glycans in Arabidopsis roots via click labeling of metabolically incorporated azido-monosaccharides
Source: BMC Plant Biol. 2016 Oct 10;16:220. doi: 10.1186/s12870-016-0907-0 (PMC5056477; doi:10.1186/s12870-016-0907-0)
Supplement: Additional file 13: — Synthesis procedures and characterization data for azido-monosaccharides. (DOCX 1338 kb) [file 12870_2016_907_MOESM13_ESM.docx]

**Additional file 13:**

**Experimental procedures, characterization data and copies of ^1^H and ^13^C NMR spectra**

**General information and methods for synthesis**

All moisture-sensitive reactions were carried out under an argon atmosphere, using oven-dried glassware, unless otherwise stated. Dichloromethane (CH_2_Cl_2_, Sigma-Aldrich, >99.8%) was purified over aluminum oxide under argon using a Pure Solv 400 solvent purification system (Innovative Technology, Amesbury, USA). Reagents were obtained from commercial sources and used without further [purification](javascript:popupOBO('CMO:0002231','C3OB40515J')) unless stated otherwise. Analytical [TLC](javascript:popupOBO('CMO:0001007','C3OB40515J')) was performed using prepared plates of [silica](http://www.chemspider.com/Chemical-Structure.22683.html) gel (Merck 60 F-254 on aluminium) and then, according to the functional groups present on the molecules, revealed with UV light or using staining reagents: [ninhydrin](http://www.chemspider.com/Chemical-Structure.9819.html) (5% in [EtOH](http://www.chemspider.com/Chemical-Structure.682.html)) for amines, H_2_SO_4_ for monosaccharides or a basic solution of KMnO_4_ (0.75% in [H_2_O](http://www.chemspider.com/Chemical-Structure.937.html)) for general staining. Merck [silica](http://www.chemspider.com/Chemical-Structure.22683.html) gel 60 (70–230 mesh) was used for [flash chromatography](javascript:popupOBO('CMO:0002582','C3OB40515J')). [^1^H NMR](javascript:popupOBO('CMO:0000593','C3OB40515J')) and [^13^C NMR spectra](javascript:popupOBO('CMO:0000837','C3OB40515J')) were recorded on a Bruker Avance III 400 spectrometer (observation of ^1^H [nucleus](javascript:popupOBO('CHEBI:33252','C3OB40515J','http://www.ebi.ac.uk/chebi/searchId.do?chebiId=33252')) 400 MHz, and of ^13^C [nucleus](javascript:popupOBO('CHEBI:33252','C3OB40515J','http://www.ebi.ac.uk/chebi/searchId.do?chebiId=33252')) 100 MHz). Chemical shifts are reported in parts per million (ppm), calibrated on the residual peak of the [solvent](javascript:popupOBO('CHEBI:46787','C3OB40515J','http://www.ebi.ac.uk/chebi/searchId.do?chebiId=46787')), whose values are referred to [tetramethylsilane](http://www.chemspider.com/Chemical-Structure.6156.html) ([TMS](javascript:popupOBO('CMO:0000531','C3OB40515J')), *δ*_TMS_ = 0), as the internal standard. [^13^C NMR spectra](javascript:popupOBO('CMO:0000837','C3OB40515J')) were performed with [proton](javascript:popupOBO('CHEBI:24636','C3OB40515J','http://www.ebi.ac.uk/chebi/searchId.do?chebiId=24636')) decoupling. Where indicated, NMR peak assignments were made using COSY and HSQC experiments.

**Synthesis of 1,3,4,4-tetra-*O*-acetyl-*N*-azidoacetyl-α,β-d-galactosamine (GalNAz) and 1,3,4,4-tetra-*O*-acetyl-*N*-azidoacetyl-α,β-d-glucosamine (GlcNAz).**

The peracetylated sugars were synthesized exactly as described by Bertozzi *et al.* [1].

**Synthesis of 1,2,3,4-tetra-*O*-acetyl-6-azido-l*-*fucose (FucAz)**

6-Azido-l-fucose (10.0 mg, 0.0448 mmol) was added to a mixture with pyridine (0.40 mL, 2.48 mmol, 100 equiv.), acetic anhydride (0.20 mL, 2.12 mmol, 400 equiv.) and 4-dimethylaminopyridine (0.1 mg, 0.008 mmol, 0.01 equiv.) The mixture was stirred at room temperature for 24 hours under an argon atmosphere. The mixture was then cooled in an ice-bath for 1 hour and then extracted with dichloromethane (20 mL, 3×). The combined organic layers were washed with sat. aq. NaHCO_3_ (20 mL) , brine (20 mL), dried (MgSO_4_) and then conc. *in vacuo*. The residue was purified by flash column chromatography (33% ethyl acetate in heptane) providing 1,2,3,4-tetra-O-acetyl-6-azido-*D*-fucose (**3**) (10.3 mg, 0.0276 mmol, 62%) as a yellow oil. **^1^H NMR** (CDCl_3_, 400 MHz): 6.31 (s, 1H, H-1), 5.34 (s, 1H, H-3), 5.25 (d, *J* = 1.2 Hz 2H, H-2, H-4,), 4.14 (t, *J* = 6.8 Hz 1H, H-5,), 3.37 (dd, *J* = 5.2, 12.8 Hz, 1 H, -CH_2_N_3_,), 3.13 (dd, *J* = 7.2, 12.8 Hz, 1H, -CH_2_N_3_), 2.09-2.03 (m, 6H, 2x –CO-CH_3_), 1.96-1.92 (m, 6H, 2x –CO-CH_3_) **^13^C NMR** (CDCl_3_, 100 MHz): 89.7 (C-1), 70.3 (C-5), 68.3 (C-3), 67.5, 66.5 (C-2, C-4), 50.5 (CH_2_N_3_), 20.9, 20.7, 20.7, 20.6 (4x CH_3_).

**Synthesis of 1,2,3-tri-*O*-acetyl-5-azido-5-deoxy*-*l-arabinofuranose (ArabAz (5))**


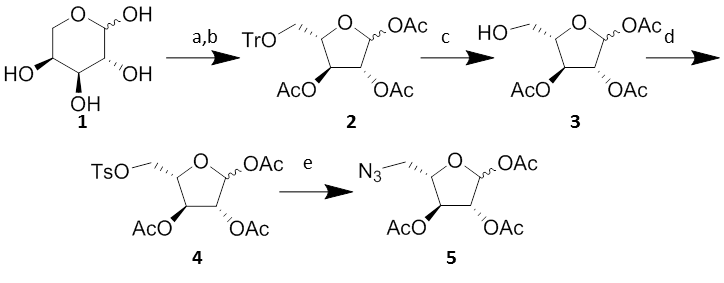


Synthesis scheme for 1,2,3-tri-*O*-acetyl-5-azido-5-deoxy*-*l-arabinofuranose ArabAz (5). Reagents and conditions: (a) Tritylchloride, pyridine, 63 h; (b) acetic anhydride, pyridine, 16 h, 19% for a,b; (c) 80% aqueous acetic acid, 100 °C, 0.5 h, 36%; (d) tosyl chloride, pyridine, dichloromethane, 48 h, 65%; (e) sodium azide, DMSO, 80 °C, 24 h, 24%.

**Synthesis of 1,2,3-tri-*O*-acetyl-5-*O-*trityl-l-arabinofuranose (2)**

l-Arabinose (**1**) (5.00 g, 33.5 mmol) was refluxed in pyridine (175 mL) until it was completely dissolved. The dissolved l-arabinose was allowed to cool to room temperature followed by the addition of trityl chloride (9.30 g, 33.5 mmol). The reaction was stirred for 63 h under an argon atmosphere. The reaction was quenched with methanol (12.5 mL). The solvents were removed *in vacuo*. The residual oily substance was dissolved in ethyl acetate (100 mL) and washed with demi water (600 mL). The organic layer was dried over MgSO_4_ and the remaining solvent was conc. *in vacuo* and the sticky product 5-*O-*trityl-l-arabinose was purified by flash column chromatography (50%, 40-60 light petrol in ethyl acetate). The product was then directly dissolved in pyridine (20 mL) and cooled to 0°C. Acetic anhydride (4.5 mL) was slowly added and the reaction was stirred for 16 h under an argon atmoshpere while allowing the reaction warming to room temperature. Afterwards the solvent was removed azeotrope (50 % toluene in ethanol, 3 ×) and dissolved in DCM (25 mL). The solution was washed with saturated NaHCO_3_ (50 mL, 2×), dried over MgSO_4_ and conc. *in vacuo*. The crystal product was purified by flash column chromatography (20 % ethyl acetate in heptane) to obtain 1,2,3, Tri-*O*-acetyl-5-*O-*trityl-L-arabinofuranose (3.10 g, 6.15 mmol) as white crystals. Yield over two steps was 19%. **^1^H NMR** (CDCl_3_, 400 MHz): 7.48-7.45 (m, 6H, ArH), 7.32-7.22 (m, 9H, ArH), 6.38 (d, *J* = 4.8 Hz, 1H, H-1β), 6.22 (s, 1H, H-1α), 5.57 (t, *J*= 6.8 Hz, 1H, H-3β), 5,34 (dd, *J* = 4.8, 7.2 Hz, 1H*,* H-2β), 5.28 (d, *J* = 4.8 Hz, 1H, H-3α), 5.20 (d, *J* = 4.0 Hz, 1H, H-2α), 4.32 (dd, *J* = 4.8, 9.4 Hz, 1H, H-4α), 4.14 (dd, *J* = 5.2, 12.6 Hz, 1H*,* , H-4β), 3.39-3.30 (m, 4H, H-5abα,β), 2.12-2.03 (6xs, 18H, 3x OAcα, 3x OAcβ). **^13^C NMR** (CDCl_3_, 100 MHz): 128.9-127.0 (15xC ArH), 99.5 (C-1α), 93.7 (C-1β), 83.6 (C-4β), 81.1 (C-2α), 80.7 (C-4β), 77.2 (C-3α), 75.6 (C-2β), 74.5 (C-3β), 64.4 (C-5β), 62.8 (C-5α), 21.1-20.5 (3x OAcα, 3x OAcβ).

**Synthesis of 1,2,3-tri-*O*-acetyl-l-arabinofuranose (3)**

1,2,3-Tri-*O*-acetyl-5-*O-*trityl-L-arabinofuranose (3.10 g, 6.15 mmol) was stirred in refluxing (100 °C) 80% aqueous acetic acid (30 mL) for 30 minutes. After 30 minutes the solution was immediately cooled in an ice bath. The by product (trityl-alcohol) was filtered off and brine (30 mL) was added. The product was extracted with DCM (10 mL, 3×) and the combined layers were washed carefully with saturated NaHCO_3_ (10 mL, 3×), dried over MgSO_4_ and conc. *in vacuo*. Purification was done by silica chromatography ( 33% ethyl acetate in heptane) to obtain 1,2,3, tri-*O*-acetyl-L-arabinofuranose (0.605 g, 2.19 mmol, 36%) as a transparent oil. **^1^H NMR** (CDCl_3_, 400 MHz): 6.38 (d, *J* = 3.6 Hz, 1 H, H-1β), 6.18 (s, 1 H, H-1α), 5.39 (d, *J* = 4.8 Hz, 1H, H-2β), 5.25 (s, 1H, H-2α), 5.12 (d, *J* = 5.2 Hz, 1H*,* H-3α), 4.23 (dd, *J* = 4.0, 8.6 Hz, 1H, H-4α), 3.90 (d, *J* = 12.4 Hz, 1H, H-4β), 3.82-3.74 (m, 4 H, , H-5abα,β), 2.15-2.02 (6x s, 18H, 3x OAcα, 3x OAcβ).**^13^C NMR** (CDCl_3_, 100 MHz): 99.2 (C-1β), 93.4 (C-1α), 85.0 (C-4α), 82.5 (C-3β), 81.0 (C-2α), 75.4 (C-3α), 74.4 (C-2β), 63.3, 61.7, 60.4 (C-4α, C-5α, C-5β), 21.0, 20.9, 20.8, 20.7, 20.6, 20.4 (3x OAcα, 3x OAcβ).

**Synthesis of 1,2,3-tri-*O*-acetyl-5-*O-*tosyl-l-arabinofuranose (4)**

1,2,3-Tri-O-acetyl-l-arabinofuranose (0.250 g, 0.905 mmol) was dissolved in 7.5 mL dry DCM and 0.75 mL pyridine followed by the addition of tosyl chloride (0.317 g, 1.6 mmol, 1.5 equiv.). The reaction was stirred for 48 hours at room temperature under an atmosphere of argon. Distilled water (20 mL) was added to the reaction mixture and the product was extract with DCM (10mL, 3×). The organic layers were combined and washed with 1M HCl (30 mL), sat. NaHCO_3_ (30 mL) and brine (30 mL) before drying with MgSO_4_ and conc. *in vacuo*. Purication was done by flash column chromatography (50% ethyl acetate in heptane) to obtain 1,2,3, tri-*O*-acetyl-5-*O*-tosyl-l-arabinofuranose (0.253 g, 0.588 mmol, 65%) as a transparent, sticky oil. **^1^H NMR** (CDCl_3_, 400 MHz): 7.79 (d, 2H, J = 7.2 Hz, ArH), 7.35 (d, 2H, J = 8.0 Hz, ArH), 6.33 (d, 1H, J = 4.0 Hz, H-1β), 6.12 (s, 1H, H-1α), 5.31 (dd, 1H, J = 7.2, 7.2 Hz), 5.16 (s, 1H, H-2α), 5.00 (d, 1H, J = 4.4 Hz, H-3α), 4.33-4.13 (m, 7H, H-3β, H-4α, H-5αa, H-5αb, H-4β, H-5αa, H-5bβ), 2.45 (s, 3H, H_3_C-ArH), 2.11-2.05 (6x s, 18 H, 3x OAcα, 3x OAcβ) **^13^C NMR** (CDCl_3_, 100 MHz): 129.8 (ArH), 128.0 (ArH), 99.3 (C-1α), 93.3 (C-1β), 80.2 (C-2α), 77.2 (C-3α), 74.3 (C-2β), 82.5, 79.3, 75.0, 69.3, 68.0 (C-4α, C-5α, C-3β, C-4β, C-5β), 20.9-20.4 (3x OAcα, 3x OAcβ).

**Synthesis of 1,2,3-tri-*O*-acetyl-5-azido-5-deoxy*-*l-arabinofuranose (ArabAz, 5)**

1,2,3-Tri-*O*-acetyl-5-*O*-tosyl-l-arabinofuranose (0.100 g, 0.24 mmol) and sodium azide (0.064 g, 0.91 mmol) were heated to 80 °C in DMSO (1.5 mL) and stirred for 24 hours. Upon cooling of the reaction after 24 hours, the reaction mixture was diluted with DCM (10 mL) and washed with demi water (40 mL). The organic layer was dried over MgSO_4_ and concentrated. Purification was done by wet column chromatography (33 % ethyl acetate in heptane). Fractions containing product were conc. *in vacuo*. The concentrated material was purified further by flash column chromatography (20 % ethyl acetate in heptane) to obtain 1,2,3-tri-*O*-acetyl-5-azido-5-deoxy-l-arabinofuranose (0.017 g, 0.056 mmol, 24%) as a colourless sticky oil. **^1^H NMR** (CDCl_3_, 400 MHz): 6.41 (d, 1H, J = 7.6 Hz, H-1β), 6.22 (s, 1H, H-1α), 5.37 (dd, 1H, J = 4.4, 8.0 Hz), 5.22 (d, 1H, J = 1.2 Hz, H-2α), 5.05 (d, 1H, J = 4.4 Hz, H-3α), 4.29 (dd, 1H, J = 4.0, 8.0 Hz, H-4α), 4.14-4.09 (m, 1H, H-3β), 3.68 (dd, 1H, J = 3.2, 13.4 Hz, H-5α), 3.49-3.43 (m, 1H, H-5β), 2.14, 2.12, 2.12, 2.11, 2.10, 2.09 ((6xs, 18H, 3x OAcα, 3x OAcβ) **^13^C NMR** (CDCl_3_, 100 MHz): 99.2 (C-1α), 93.5 (C-1β), 84.1 (C-4α), 80.8 (C-3β), 80.6 (C-2α), 77.2 (C-3α), 74.8 (C-2β), 75.1 (C-4β), 53.0 (C-5β), 51.3 (C-5α), 21.0-20.4 (3x OAcα, 3x OAcβ).

**Adapted synthesis of 3-hydroxymethyl-2-methyl-trimethylsilylcyclopropene (6)**

Synthesis scheme for 3-hydroxymethyl-2-methyl-trimethylsilylcyclopropene (6). Reagents and conditions: (a) Rhodium(II) acetate dimer, DCM, 12 h; (b) DIBAL-H, Et_2_O, 2 h, 96% over two steps. Intermediate 6 was converted into Ac_4_GlcCyc using previously reported procedures [2, 3].

An oven-dried 50 mL round bottom flask was placed under vacuum and backfilled with argon. Rhodium(II) acetate dimer (15 mg, 0.034 mmol) was added to the flask under an argon flow. Next, tri-methyl silyl propyne (4 mL, 27.01 mmol) was added and the resulting suspension was stirred slowly. A solution of ethyl diazoacetate (1.1 mL, 13% in DCM, 9.06 mmol) was diluted with 15 mL anhydrous DCM into a separate oven dried round bottom flask under argon. This solution was transferred to a 20 mL syringe and very slowly added over roughly 12 h to the roundbottom flask containing the suspension using a syringe pump (20 μL/min, NE-1000 Multi-Phaser™) under a gentle argon flow. During the addition period the observed colour of the reaction mixture went from a colourless solution with a dark purple solid to a dark green reaction mixture and towards the end to a yellow-greenish reaction mixture. After the addition, the rhodium catalyst was separated by passing the reaction mixture over a silica gel plug (40 mL) and flushing with 100 mL DCM. The collected eluent was gently concentrated under a reduced pressure at room temperature to yield the crude intermediate as an oil that was used in the next reaction without further purification (R*_f_* 0.8 in 3:7 EtOAc:PE, KMnO_4_ stain).

Di-isobutyl aluminium-hydride (18 mL, 1M in THF, 18 mmol) was added to anhydrous diethyl ether (15 mL) in an oven-dried roundbottom flask and cooled to 4 °C under an argon atmosphere. The complete crude ethyl TMS-cyclopropene acetate product from the previous reaction was dissolved in anhydrous diethyl ether (5 mL) and added dropwise over 5 min. to the DIBAL-H containing flask. The reaction mixture was stirred for 2 hours at 4 °C under an argon atmosphere. Conversion to **6** was monitored by TLC (R*_f_* ethyl TMS-cyclopropene acetate = 0.8; Rf **6** = 0.6, in 30:70 EtOAc: PE, KMnO_4_ stain), and shown to be incomplete. Accordingly, additional DIBAL-H (7 mL; 7 mmol) was slowly added to the reaction mixture at 4 °C, after which TLC indicated complete conversion in under ten minutes. The reaction mixture was quenched by addition of a saturated aqueous solution of Rochelle’s salt (300 mL) under vigorous stirring. A white gel formed in the aqueous layer. The biphasic system was separated and the water layer was extracted with EtOAc (50 mL, 3×). The combined organic layers were dried (NaSO_4_), filtered and gently evaporated under reduced pressure at room temperature. The resulting residue was purified using flash silica gel column chromatography (eluent: 10 → 30% EtOAc in PE) to provide **6** in 96% yield over two steps (1.37 g, 8.76 mmol) as a pale yellow liquid with a pine-like odour. **^1^H NMR** (CDCl_3_, 400 MHz): 3.46 (q, 2H), 2.19 (s, 3H), 1.54 (t, 1H), 0.15 (s, 9H). Characterization is in agreement with previously reported ^1^H NMR spectrum [2].

**NMR spectra of azido-monosaccharides**

**References**

[1] Laughlin, S.T., Agard, N.J., Baskin, J.M., Carrico, I.S., Chang, P.V., Ganguli, A.S., Hangauer, M.J., Lo, A., Prescher, J.A., and Bertozzi, C.R., *Metabolic Labeling of Glycans with Azido Sugars for Visualization and Glycoproteomics*, in *Methods Enzymol.* 2006, Academic Press. p. 230-50.

[2] Patterson, D.M., Jones, K.A., and Prescher, J.A. Improved cyclopropene reporters for probing protein glycosylation. *Molecular BioSystems.* 2014; *10*:1693-97

[3] Späte, A.-K., Bußkamp, H., Niederwieser, A., Schart, V.F., Marx, A., and Wittmann, V. Rapid Labeling of Metabolically Engineered Cell-Surface Glycoconjugates with a Carbamate-Linked Cyclopropene Reporter. *Bioconjugate Chem.* 2014; *25*:147-54
