# Supplementary material for: Direct imaging of glycans in Arabidopsis roots via click labeling of metabolically incorporated azido-monosaccharides
Source: BMC Plant Biol. 2016 Oct 10;16:220. doi: 10.1186/s12870-016-0907-0 (PMC5056477; doi:10.1186/s12870-016-0907-0)
Supplement: Additional file 14: — Figures in high resolution. (ZIP 22425 kb) [file 12870_2016_907_MOESM14_ESM.zip › Figure 2.pdf]

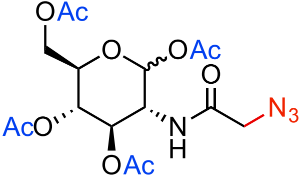

Ac$_4$GlcNAz

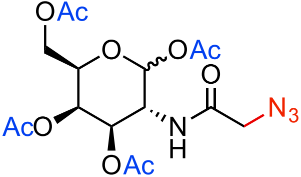

Ac$_4$GalNAz

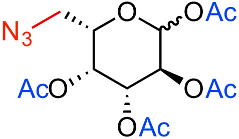

Ac$_4$FucAz

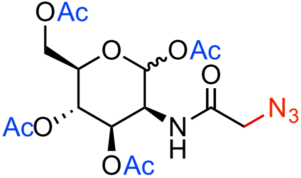

Ac$_4$ManNAz

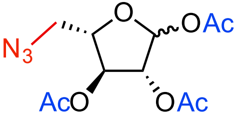

Ac$_3$ArabAz

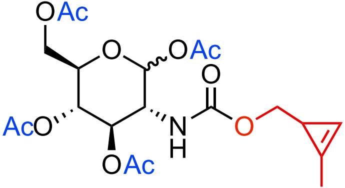

Ac$_4$GlcNCyc
