# Supplementary figures and images for: Direct imaging of glycans in Arabidopsis roots via click labeling of metabolically incorporated azido-monosaccharides
Source: BMC Plant Biol. 2016 Oct 10;16:220. doi: 10.1186/s12870-016-0907-0 (PMC5056477; doi:10.1186/s12870-016-0907-0)

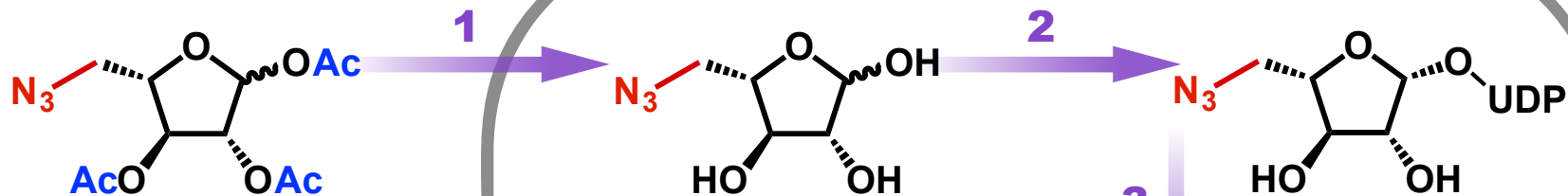

Supplement: Additional file 14: — Figures in high resolution. (ZIP 22425 kb) [file 12870_2016_907_MOESM14_ESM.zip › Figure 1.pdf]

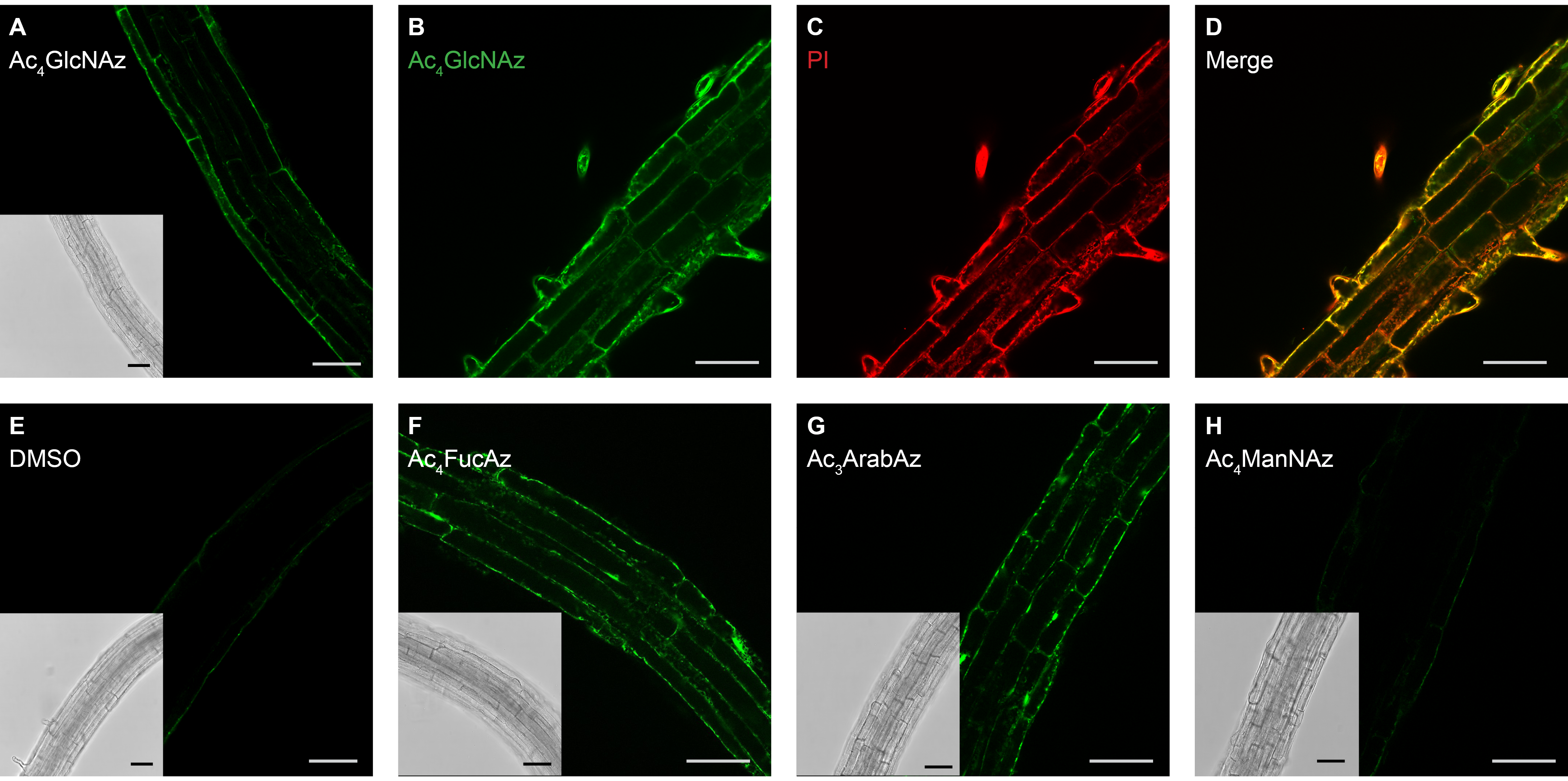

Supplement: Additional file 14: — Figures in high resolution. (ZIP 22425 kb) [file 12870_2016_907_MOESM14_ESM.zip › Figure 3.png]

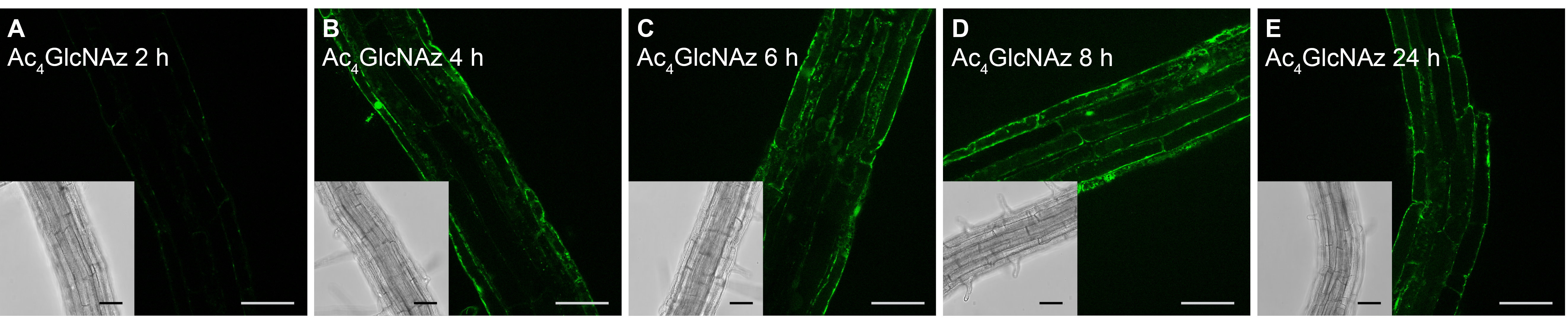

Supplement: Additional file 14: — Figures in high resolution. (ZIP 22425 kb) [file 12870_2016_907_MOESM14_ESM.zip › Figure 4.png]

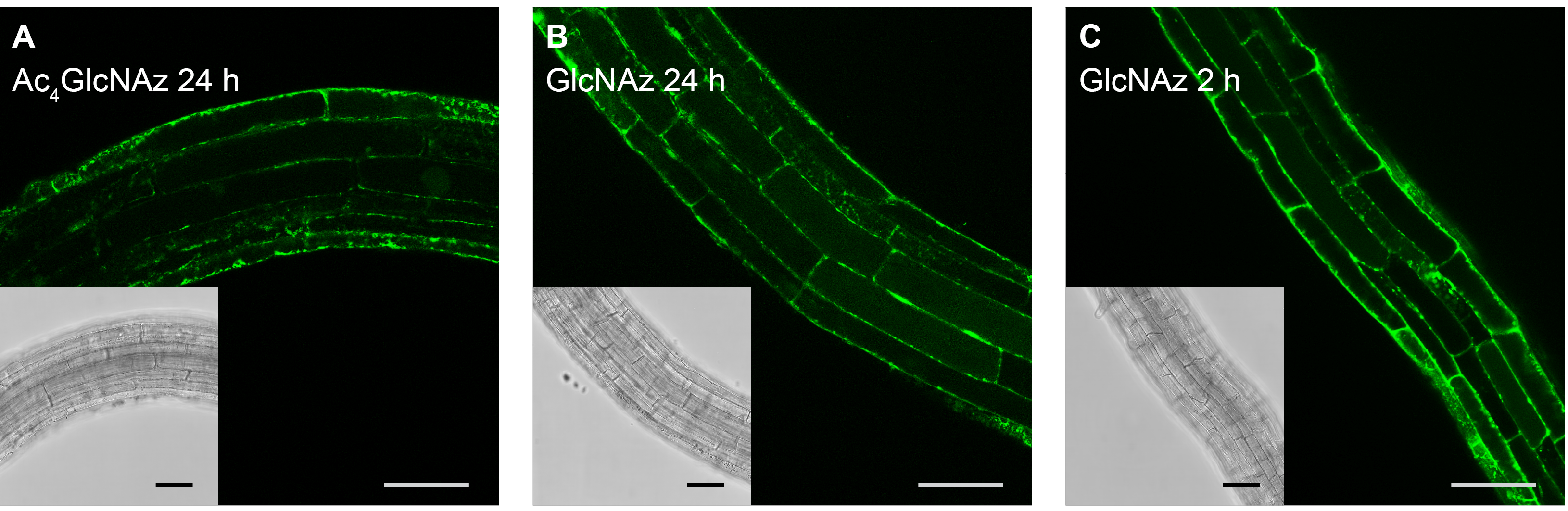

Supplement: Additional file 14: — Figures in high resolution. (ZIP 22425 kb) [file 12870_2016_907_MOESM14_ESM.zip › Figure 5.png]

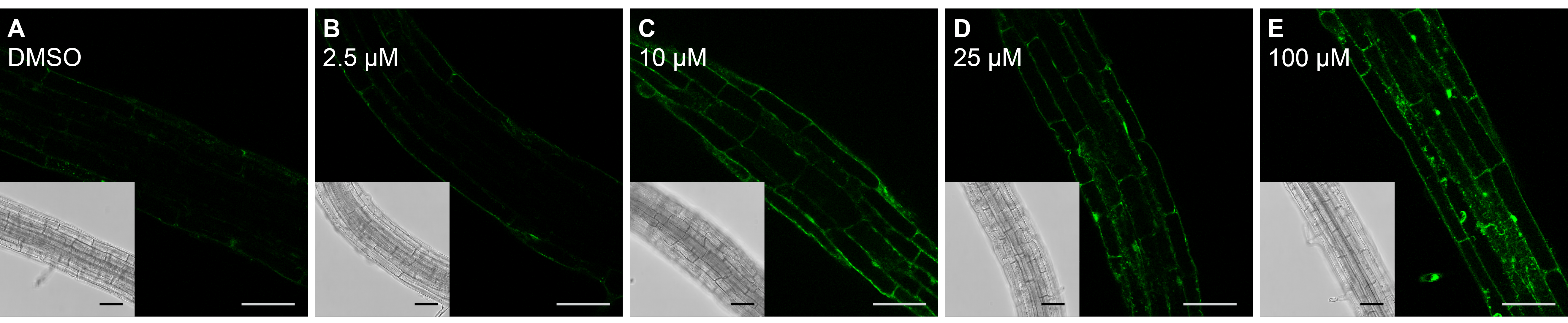

Supplement: Additional file 14: — Figures in high resolution. (ZIP 22425 kb) [file 12870_2016_907_MOESM14_ESM.zip › Figure 6.png]

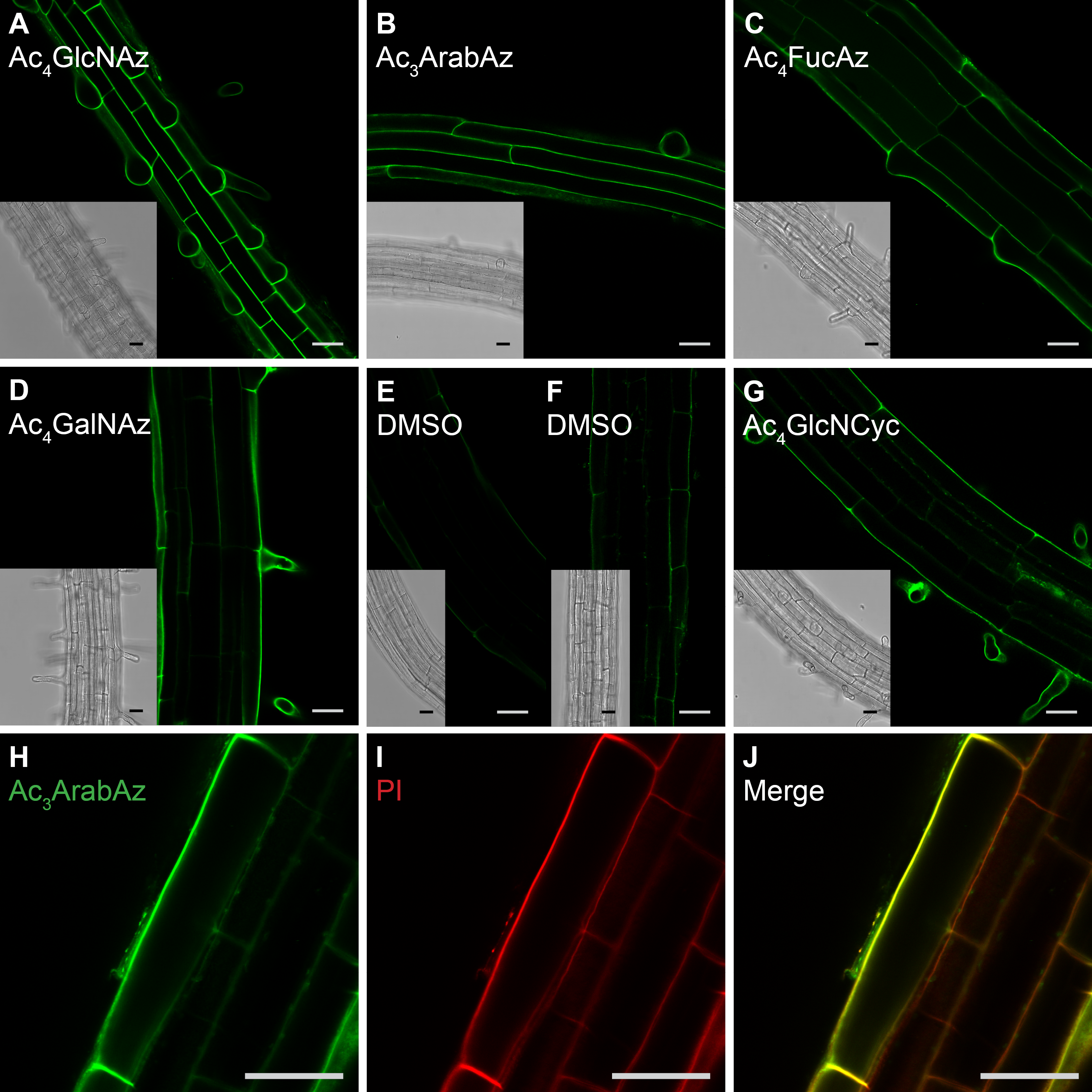

Supplement: Additional file 14: — Figures in high resolution. (ZIP 22425 kb) [file 12870_2016_907_MOESM14_ESM.zip › Figure 7.png]
